# Facile Preparation of a Glycopolymer Library by PET-RAFT Polymerization for Screening the Polymer Structures of GM1 Mimics

**DOI:** 10.1021/acsomega.2c00719

**Published:** 2022-04-06

**Authors:** Masanori Nagao, Yuri Kimoto, Yu Hoshino, Yoshiko Miura

**Affiliations:** Department of Chemical Engineering, Kyushu University, 744 Motooka Nishi-ku, Fukuoka 819-0395, Japan

## Abstract

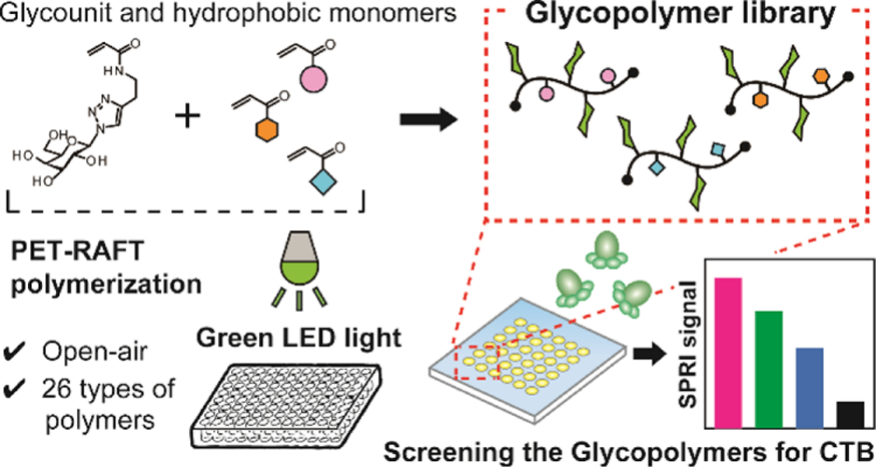

Commercialized oligosaccharides such as GM1 are useful for biological applications but generally expensive. Thus, facile access to an effective alternative is desired. Glycopolymers displaying both carbohydrate and hydrophobic units are promising materials as alternatives to oligosaccharides. Prediction of the appropriate polymer structure as an oligosaccharide mimic is difficult, and screening of the many candidates (glycopolymer library) is required. However, repeating polymerization manipulation for each polymer sample to prepare the glycopolymer library is time-consuming. Herein, we report a facile preparation of the glycopolymer library of GM1 mimics by photoinduced electron/energy transfer-reversible addition–fragmentation chain-transfer (PET-RAFT) polymerization. Glycopolymers displaying galactose units were synthesized in various ratios of hydrophobic acrylamide derivatives. The synthesized glycopolymers were immobilized on a gold surface, and the interactions with cholera toxin B subunits (CTB) were analyzed using surface plasmon resonance imaging (SPRI). The screening by SPRI revealed the correlation between the log *P* values of the hydrophobic monomers and the interactions of the glycopolymers with CTB, and the appropriate polymer structure as a GM1 mimic was determined. The combination of the one-time preparation and the fast screening of the glycopolymer library provides a new strategy to access the synthetic materials for critical biomolecular recognition.

## Introduction

Carbohydrates on the cell surface are involved in biological phenomena such as pathogen infections through carbohydrate–protein interactions.^1,2^ In particular, oligosaccharides play an important role in the living system. For example, GM1 ganglioside, which has galactose (Gal) and neuraminic acid (Neu5Ac) residues as the two nonreducing ends, is related to cell differentiation, neurodegenerative diseases, and pathogen infection.^3,4^ Although bioactive oligosaccharides are demanded for various biological applications, commercialized oligosaccharides are generally expensive due to the difficulty of the total synthesis and of the extraction from bioreactors. The inconvenient access to the oligosaccharides has motivated researchers to seek effective and inexpensive alternatives.

Synthetic glycopolymers have gathered attention as effective alternatives to oligosaccharides.^5,6^ Glycopolymers, which are macromolecules displaying multiple glycounits, have been developed as glycomimetics for the interaction with carbohydrate recognition proteins (lectins).^7,8^ To overcome the weakness of the monovalent carbohydrate–protein interaction, glycopolymers multivalently bind to lectins, amplifying the total interaction (the cluster glycoside effect).^9^ Our group has recently demonstrated a synthetic approach for GM1 mimics, the “carbohydrate module method”.^10−12^ GM1 binds to cholera toxin B subunits (CTB) through the interaction between the nonreducing ends (Gal and Neu5Ac units) and the carbohydrate recognition domain of CTB. In particular, the Gal unit binds to the deep part of the domain and mainly contributes to the interaction with CTB.^13^ In the “carbohydrate module method”, the critical part of the GM1 structure for molecular recognition is reconstructed by the radical polymerization of glycomonomers. The glycopolymers displaying both Gal and Neu5Ac units in a certain ratio showed strong interaction with CTB. Another strategy to gain access to GM1 mimics was presented by Gibson and co-workers.^14^ They prepared a series of glycopolymers containing galactose monomers with adjacent hydrophobic groups and demonstrated the improvement in the CTB recognition of the glycopolymers. This pioneering work suggests that hydrophobic groups can interact with the part of the binding pockets of CTB where sialic acid of GM1 interacts. The glycopolymers mimicking the functions of the oligosaccharides exhibit the cluster glycoside effect and have been utilized in biological applications.

Although the glycopolymers are promising materials as oligosaccharide alternatives, the optimal design of the polymer structures for a specific target is difficult because the binding mode of the glycopolymers to the target lectins is not completely predictable. Thus, screening of the polymer structures from many candidates (a glycopolymer library) is necessary to determine the effective polymer composition.^15^ However, synthesis of the glycopolymers requires heating and degassing, making the library preparation time-consuming. Boyer and co-workers developed oxygen-tolerance radical polymerization techniques (photoinduced electron/energy transfer reversible addition–fragmentation chain-transfer polymerization; PET-RAFT polymerization) and enabled the preparation of well-defined synthetic polymers in an open-air environment.^16,17^ This excellent technique has been applied to the facile preparation of the polymer library^18−20^ and polymer brushes on the surface.^21,22^ Furthermore, the high selectivity of PET-RAFT polymerization provided the discrete materials through radical reaction,^23,24^ and the reaction selectivity was controlled by the colors of the irradiated light.^25^

Herein, we report the facile preparation of the glycopolymer library of GM1 mimics by PET-RAFT polymerization. Glycopolymers displaying Gal units were synthesized by co-polymerizing the glycomonomer and hydrophobic acrylamide derivatives in various ratios, aiming at improvement of the binding affinity for CTB by the cooperative effect of the hydrophobic groups.^14,15^ The synthesized glycopolymers were immobilized on a gold surface, and the interactions with CTB were analyzed using surface plasmon resonance imaging (SPRI). The SPRI screening of the glycopolymer library unraveled the correlation between the characters of hydrophobic groups and the interactions of the glycopolymers with CTB.

## Results and Discussion

### Preparation of a Glycopolymer Library by PET-RAFT Polymerization

**Figure 1:**
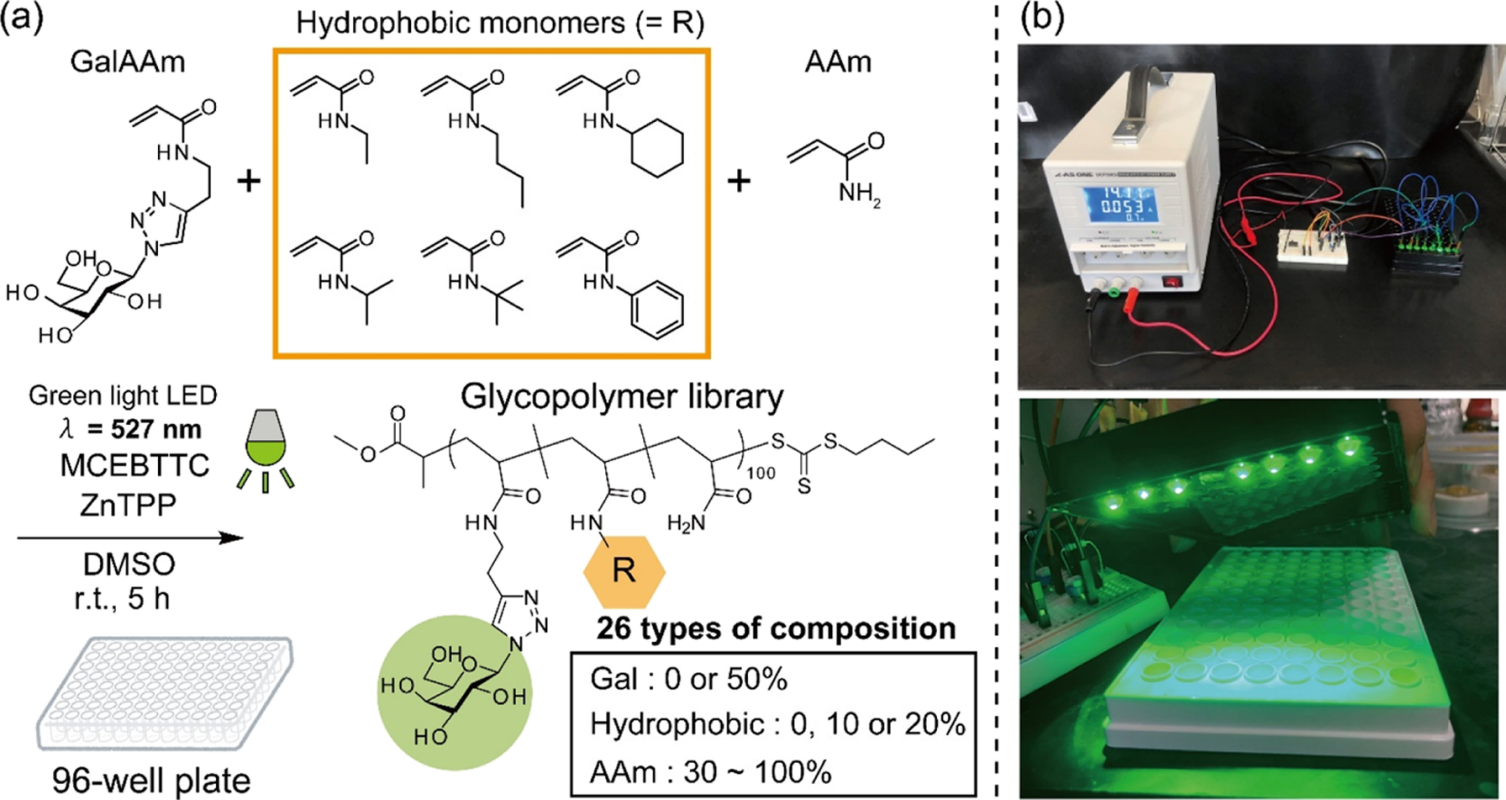
(a) Synthetic scheme of the glycopolymer library. (b) Picture of the PET-RAFT polymerization equipment under the open-air condition.

Glycopolymers with various monomer compositions (glycopolymer library) were prepared by PET-RAFT polymerization in an open-air condition. Galactose acrylamide (GalAAm), acrylamide (AAm), and each hydrophobic acrylamide derivative (EthylAAm, NIPAm, TBAm, ButylAAm, CyHexAAm, and PhAAm) were mixed in various compositions, and a library with 26 types of the mixture was prepared (Figure 1a). AAm, which is inert for biomolecular recognition, was used as the hydrophilic spacer units in the polymer structures. To assure the solubility of the objective polymers in an aqueous buffer solution, the feed ratio of the hydrophobic monomers was set at no more than 20%. The monomer concentration [M] was fixed at 0.5 M. The monomers, RAFT agent [methyl 2-(butylthiocarbonothioylthio)propanoate, MCEBTTC], and photocatalyst [zinc(II)tetraphenyl porphyrin, ZnTPP] were mixed at a ratio of 100:1:0.02 in dimethyl sulfoxide (DMSO) (200 μL). Each mixture was added to a 96-well plate and irradiated with green light-emitting diode (LED) light (λ = 527 nm) for 5 h at room temperature (Figure 1b). The wavelength of the irradiation light was selected based on the absorption peak of ZnTPP (Figure S1). The monomer conversion and molecular weights were determined by proton nuclear magnetic resonance (^1^H NMR) and size exclusion chromatography (SEC) analysis, respectively (Table 1). In most cases, the monomer conversions were over 90%, and the polymerization proceeded successfully under the open-air condition. The relative molecular weights (*M*_n_) of the polymers without glycounits were around 5000 g/mol, and these values were almost half of those of the glycopolymers (around 10,000 g/mol), even though the target degree of polymerization was fixed as 100 for all the polymers. This suggested that the glycopolymers extended in DMSO due to the bulky carbohydrate structures in the side chains. Although the dispersity (*M*_w_/*M*_n_) values of the glycopolymers were relatively broad as a RAFT polymerization system (<1.60), the values were still narrower than for a free-radical polymerization system (Table 1). These results demonstrated that the PET-RAFT polymerization enabled the one-time preparation of 26 types of glycopolymers without degassing of the solutions. The trithiocarbonate terminals of the synthesized glycopolymers were sequentially reduced to thiol groups by adding sodium borohydride, and the polymers were purified by ultrafiltration. The compositions of the glycopolymers were characterized by ^1^H NMR, and the incorporated monomer ratios corresponded to the feed monomer ratios (Table 1). The ultraviolet (UV)–visible measurement of the polymerization solution was taken before ultrafiltration, and the absorbance peak of trithiocarbonate at 310 nm disappeared after the reduction treatment (Figure S2). This indicated that the reduction of the trithiocarbonate completely proceeded.

**Table 1:** Glycopolymer Library Prepared by PET-RAFT Polymerization^a^

polymer^b^	GalAAm (%)	hydrophobic monomer (%)	AAm (%)	Conv. (%)^c^	Gal unit^c^ (%)	hydrophobic unit^c^ (%)	*M*_n_^d^ (g/mol)	*M*_w_/*M*_n_^d^
G50	50	0	50	97	45	0	7600	1.55
G50E10	50	10	40	97	40	3	9200	1.36
G50E20	50	20	30	99	48	22	9900	1.42
G50N10	50	10	40	97	52	10	7600	1.40
G50N20	50	20	30	98	43	21	8700	1.37
G50B10	50	10	40	96	49	8	9800	1.46
G50B20	50	20	30	98	43	17	10,700	1.52
G50T10	50	10	40	96	44	10	10,400	1.32
G50T20	50	20	30	98	47	18	14,300	1.54
G50C10	50	10	40	95	46	6	11,400	1.59
G50C20	50	20	30	99	45	18	13,600	1.44
G50P10	50	10	40	97	48	10	11,100	1.56
G50P20	50	20	30	99	46	20	10,900	1.60
G0	0	0	100	95	0	0	5400	1.20
G0E10	0	10	90	89	0	9	5000	1.44
G0E20	0	20	80	89	0	19	5400	1.45
G0N10	0	10	90	90	0	3	4800	1.40
G0N20	0	20	80	88	0	7	4300	1.29
G0B10	0	10	90	91	0	8	6000	1.30
G0B20	0	20	80	90	0	18	5000	1.54
G0T10	0	10	90	88	0	9	5100	1.47
G0T20	0	20	80	90	0	18	5600	1.41
G0C10	0	10	90	91	0	9	5000	1.48
G0C20	0	20	80	89	0	18	5000	1.26
G0P10	0	10	90	92	0	9	5400	1.34
G0P20	0	20	80	92	0	17	5400	1.36

a The ratio of [monomer]/[RAFT]/[ZnTPP] = 100:1:0.02.

b G, E, N, T, B, C, and P represent GalAAm, EthylAAm, NIPAm, TBAm, ButylAAm, CyHexAAm, and PhAAm, respectively.

c monomer conversion and incorporated monomer ratio were determined by ^1^H NMR.

d The relative molecular weight (*M*_n_) and dispersity (*M*_w_/*M*_n_) values were determined by SEC analysis calibrated with a polystyrene standard. The eluent was DMSO with 10 mM LiBr.

### Immobilization of the Synthesized Polymers onto the Au Surface of an SPRI Chip

**Figure 2:**
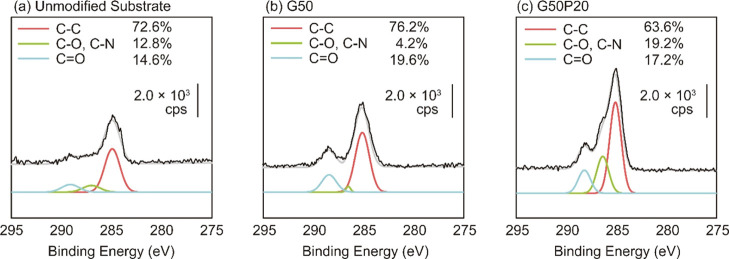
XPS C(1s) spectrum of the unmodified substrate (a), G50 (b)-, and G50P20 (c)-immobilized gold surface. The percentage values in the spectra indicate the ratio of the integral values of each divided peak.

The synthesized polymers with thiol groups were immobilized on the gold surface of the SPRI chip through the Au–thiol interaction. The immobilization of the polymers was confirmed by an X-ray photoelectron spectroscopy measurement (XPS measurement). After the immobilization of G50 and G50P20, the peaks of C–C (284.8 eV), C–O and C–N (286.7 eV), and C=O (287.3 eV) bonds were increased compared to the unmodified surface (Figure 2). The increased peak at 287.3 eV derived from the C=O bonds of the amide structure indicated the successful immobilization of the glycopolymers.

### Screening of the Glycopolymer Library by SPRI Measurement

**Figure 3:**
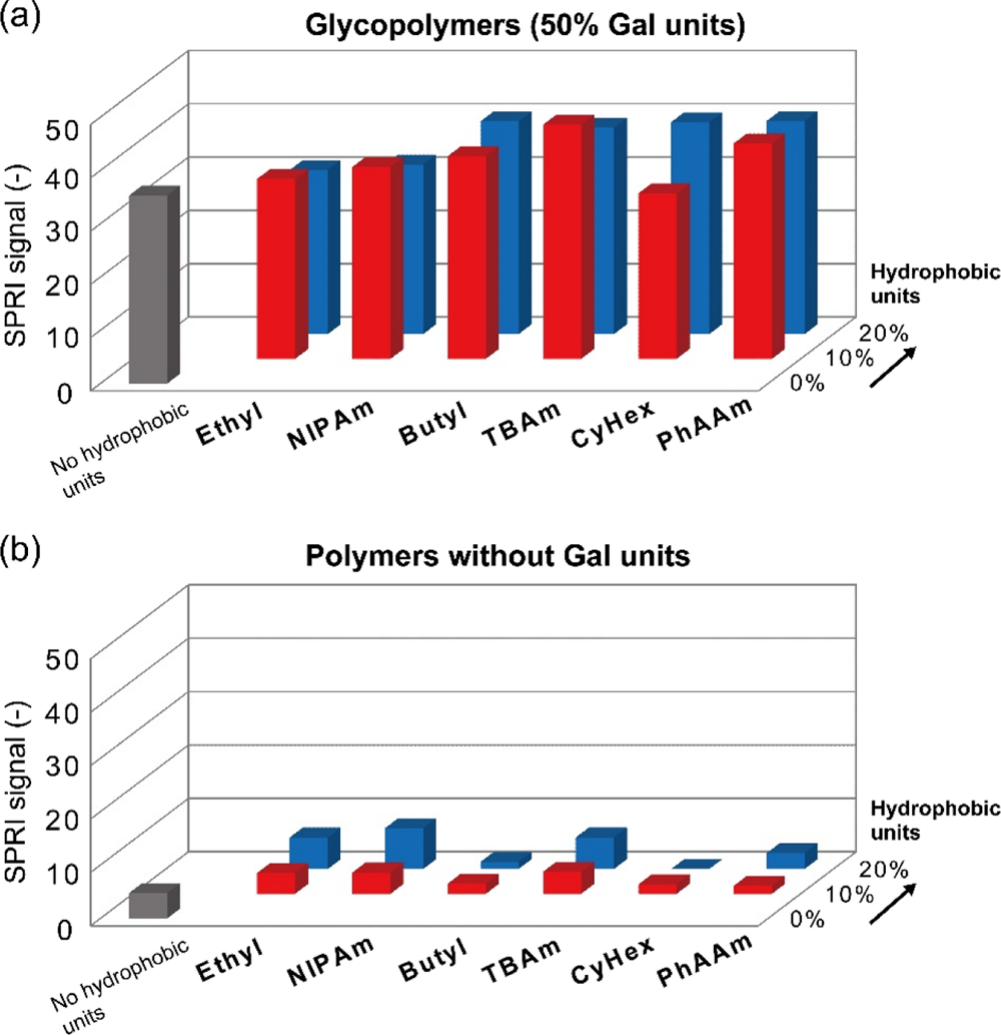
SPRI signals of the polymer-immobilized surfaces with CTB (500 nM). (a) Glycopolymers with Gal units and the (b) polymers without glycounits.

To evaluate the functions of the glycopolymers as GM1 mimics, the interactions between the glycopolymers and CTB were analyzed by SPRI measurement. After equilibration with phosphate-buffered saline (PBS) (−) buffer (0.1 mL/min for 60 min), a CTB solution (500 nM) was added. The glycopolymer-immobilized surface showed SPRI signals of more than 30, indicating the CTB’s binding to the glycopolymers (Figure 3a). Conversely, the polymer surfaces without glycounits showed signals lower than 10, indicating that CTB was not adsorbed on the surfaces (Figure 3b). These results demonstrated that CTB recognized the galactose units of the synthesized glycopolymers, which corresponded to the fact that the galactose unit of GM1 critically contributes to the interaction with CTB.^26−28^ Furthermore, the nonspecific adsorption of bovine serum albumin (BSA) was not observed for the glycopolymer-immobilized surface, and this assured that the Au surfaces were fully covered by the synthesized polymers through the Au–thiol interaction (Figure S3).^29^

**Figure 4:**
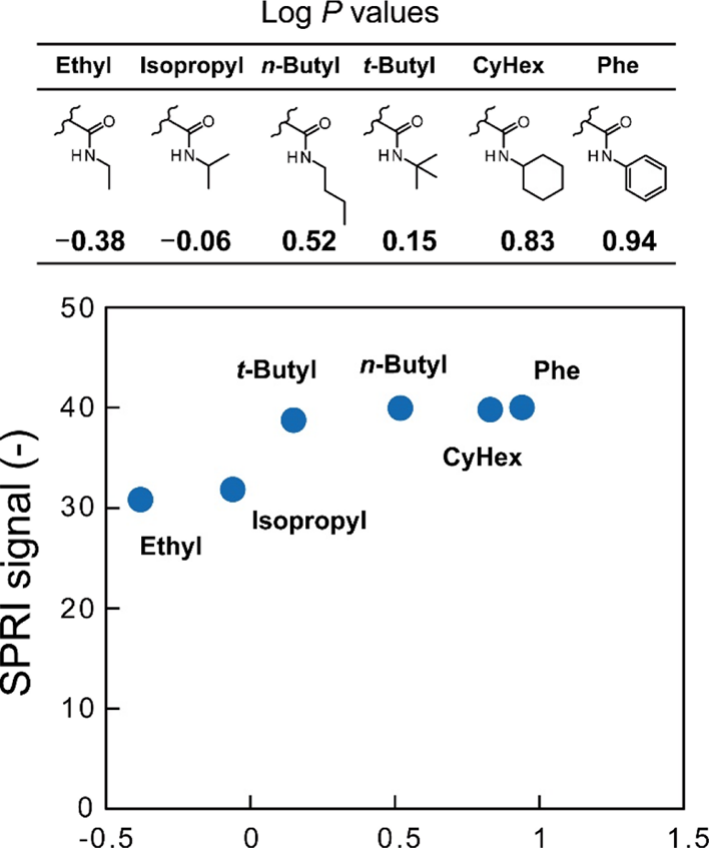
Plots of the SPRI signals of the glycopolymer-immobilized surface (20% hydrophobic units) and the log *P* values of the hydrophobic groups. The log *P* values were estimated using ChemDraw software.

The glycopolymers showed different SPRI signals based both on the structure and ratios of the hydrophobic units. The SPRI signals of G50B10, G50B20, G50T10, G50T20, G50CyHex20, GP50P10, and G50P20 for the CTB solution were 38.1, 39.9, 44.0, 38.7, 39.8, 40.5, and 40.0, respectively, and these values were higher than that of G50 (35.3). This indicated that the hydrophobic units in the polymer structures enhanced the interactions with CTB and that the contributions were different for the functional groups. The hydrophobicity of the functional groups of the co-monomers was quantified by the log *P* value, and the SPRI signals of the glycopolymers with 20% hydrophobic monomers for CTB showed a correlation with the log *P* values (Figure 4).^15^ This suggested that the hydrophobic groups with log *P* values higher than 0.15 exhibited a cooperative effect in binding to the pockets of CTB, resulting in enhanced interactions on the glycopolymer-immobilized surface. However, the correlation between the SPRI signals and the log *P* values was not observed for a lower co-monomer incorporation ratio (10%) due to the insufficient amount for the interactions with the binding pockets of CTB (Figure S4). To evaluate the binding affinity of the glycopolymers to CTB, the apparent binding constants (*K*_a_) were measured. The *K*_a_ of the G50T10-immobilized surface was 1.63 × 10^7^ M^–1^. This value was still relatively high for a GM1 mimetic polymer, even though the GM1-immobilized surface shows the higher binding constant (*K*_a_ = 10^10^ M^–1^).^26^

## Conclusions

In this report, the facile preparation of the glycopolymer library was realized by PET-RAFT polymerization in an open-air condition, and 26 types of candidates for glycomimicry of the GM1 ganglioside were obtained. The hydrophobic monomers with various log *P* values were incorporated into the glycopolymers to enhance the interaction with the target CTB. The screening of the SPRI measurements revealed that the amount of CTB adsorbed on the glycopolymer-immobilized surfaces was dependent on the log *P* values of the co-polymerized hydrophobic monomers. As a result, the synthesized glycopolymers displaying both of the galactose units and the hydrophobic units selectively interacted with CTB and exhibited the same biological function as GM1 mimics. Because the appropriate structures of glycopolymers as oligosaccharide mimics are unpredictable, the facile preparation of the polymer library and sequential screening using SPRI measurements is a useful way to seek the effective alternatives of oligosaccharides for practical biological applications.

## Experimental Section

### Preparation of a Glycopolymer Library by PET-RAFT Polymerization

The mixture of glycomonomer (GalAAm), AAm, and each hydrophobic monomer (EthylAAm, NIPAm, TBAm, ButylAAm, CyHexAAm, and PhAAm) were polymerized using PET-RAFT polymerization as per previous studies.^15^ Setting the monomer concentration to 0.5 M, the monomers, RAFT agent (MCEBTTC), and photocatalyst (ZnTPP) were dissolved in DMSO (200 μL) at a molar ratio of 100:1:0.02. The mixtures of various monomer compositions were put in wells of the 96-well plate and were irradiated by LED lights (λ = 527 nm) at room temperature for 5 h. The conversion rates were determined by ^1^H NMR, and the relative molecular weights and polydispersity index were calculated by gel permeation chromatography analysis.

### Reduction of Trithiocarbonate Terminals of Glycopolymers and Purification

A DMSO solution of sodium borohydride (1 g/L, 20 μL) was added to each well (200 μL of the glycopolymer solution), and the mixture was incubated for 12 h at room temperature. The glycopolymers were extracted from DMSO by precipitation in acetone (30 mL) twice. Then, the precipitates were dissolved in water (1 mL). The solution was added to an ultrafiltration filter (MWCO: 3000), and purification was repeated three times by a centrifuge (14,000*g*, 15 min). The filter tip was turned over and the sample was collected again in a centrifuge (1000*g*, 10 min) and then freeze-dried to obtain solid glycopolymer samples.

### SPRI Chip Preparation and SPRI Measurement

The obtained glycopolymers were immobilized on gold surfaces for SPRI measurement in the same procedure as in the previous literature. Prepared chips were set in the SPRI chip cell. Before the protein solution in PBS was flowed into the cell, 10 mM PBS (pH 7.4, 137 mM NaCl and 2.68 mM KCl) was flowed through (0.1 mL/min) until the SPR reflectivity became stable. The SPR reflectivity change (SPRI signal) was measured after injecting the protein solution (CTB or BSA) at a flow rate of 0.1 mL/min for 1 h.

## Supporting Information Available

Additional details on the experimental methods, spectra of ^1^H NMR and UV absorbance, and results of the SPRI measurement (PDF)
