# Supplementary material for: Facile Preparation of a Glycopolymer Library by PET-RAFT Polymerization for Screening the Polymer Structures of GM1 Mimics
Source: ACS Omega. 2022 Apr 6;7(15):13254–9. doi: 10.1021/acsomega.2c00719 (PMC9026043; doi:10.1021/acsomega.2c00719)
Supplement: Supplementary file 1 — ao2c00719_si_001.pdf [file ao2c00719_si_001.pdf]

Supporting information

Facile preparation of a glycopolymer library by PET-RAFT polymerization for screening the polymer structures of GM1 mimics

Masanori Nagao,[1] Yuri Kimoto,[1] Yu Hoshino[1] and Yoshiko Miura[1]*

[1]Department of Chemical Engineering, Kyushu University, 744 Motooka, Nishi-ku, Fukuoka 819-0395, Japan.

E-mail: miuray@chem-eng.kyushu-u.ac.jp

Materials

The following chemical agents were purchased from commercial sources, and were used as received unless otherwise indicated: *N,N*-diisopropylethylamine (DIPEA), cyclohexylamine, and acryloyl chloride were purchased from Tokyo Chemical Industry (Tokyo, Japan). Pottasium hydroxide (KOH), sodium borohydride ascorbic acid, and sodium ascorbate (L-Asc-Na) were purchased from Kanto Chemical (Tokyo, Japan). Acrylamide (AAm), butylamine, copper(II) sulfate pentahydrate ($CuSO_4$), Eosin Y, *N*-isopropylacrylamide (NIPAm), and hydrochloric acid (HCl) were purchased from Wako Pure Chemical Industries (Osaka, Japan). *N-tert*-Butylacrylamide (TBAm) was purchased from MRC UNITEC Co., Ltd. (Aomori, Japan). Dimethyl sulfoxide (DMSO) was purchased from Kishida chemical (Osaka, Japan). *N*-Ethylacrylamide, *N*-phenylacrylamide (PhAAm), zinc(II) tetraphenylporphyrin (ZnTPP), Sigmacote®, bovine serum albumin, and cholera toxin B subunit (CTB) was purchased from Sigma Aldrich (St. Louis, USA). Amicon Ultra-0.5 PLBC Ultracel-3 membrane (3 kDa) was purchased by Mealk (Darmstadt, Germany). LED source (L5-G2530-20000) (λ_{max} = 525 nm, 9.7 mW/cm^2) was purchased by Linkman (Japan, Fukui). AAm and NIPAm were purified prior to use by recrystallization from MeOH or mixture of benzene/*n*-hexane, respectively. 3-Butynyl acrylamide (BtnAAm),[1] galactose azide,[2] tris(benzyltriazolyl-methyl)amine (TBTA),[3] and a RAFT agent (MCEBTTC)[4] was synthesized referring the previous reports.

Characterization

Proton and carbon nuclear resonance (^{1}H NMR) spectra were recorded on a JEOL-ECP400 spectrometer (JEOL, Tokyo, Japan) using DMSO-d_6, or D_2O as a deuterated solvent. Partition coefficients (Log*P*) were estimated using ChemDraw (ver.17.1). Size exclusion chromatography (SEC) with DMSO was performed on a JASCO DG-2080-53 degasser equipped with a JASCO PU-2080 Plus pump (JASCO Co., Tokyo, Japan), a Shodex OH pak SB-G guard column, a Shodex GPU pak LF-804 HQ column (Showa Denko, Tokyo, Japan), and a JASCO RI-2031 Plus RI detector. SEC analyses were performed by injecting 20 μL of a polymer solution (2.5 g/L) in DMSO buffer with 10 mM LiBr. The SEC system was calibrated using a polystyrene standard (Shodex). All the samples for SEC were previously filtered through a 0.45 μm filter. The buffer solution was also used as the eluent at a flow rate of 0.5 mL/min. UV-vis spectra were recorded at room temperature using an Agilent 8453 spectrophotometer (Agilent Technologies, Santa Clara, CA, USA). For the analysis of the glycopolymer-immobilized gold surfaces, x-ray photoelectron spectroscopy (XPS) (AXISultra; Shimazu/Kratos, Kyoto, Japan) measurement was performed. All SPRI measurements were performed using MultiSPRinter (Toyobo Co., Ltd., Osaka, Japan).

Setup of the equipment for PET-RAFT polymerization at open-air condition.

The equipment for PET-RAFT polymerization was assembled using a regulated power supply, a circuit board, and LEDs. Each LED bulb was fitted into a 96-well plate with a hole diameter of 4.5 mm for each well to serve as a light source. Two circuits, where four LEDs were connected in series, were connected in parallel to the power supply. The voltage and current of the regulated power supply were set as 14 V and 0.05 A, respectively.

Synthesis of galactose acrylamide (GalAAm)

TBTA (265 mg, 0.5 mmol), galactose azide (1.02 g, 5.0 mmol), BtnAAm (615 mg, 5.0 mmol), and $CuSO_4$ (80 mg, 0.5 mmol) were dissolved in MeOH (25 mL) / H_2O (25 mL) mixture. The oxygen was removed by bubbling nitrogen. L-Asc-Na (200 mg, 1.0 mmol) was added and stirred at 30 °C for 24 h under nitrogen atmosphere. The solution was concentrated under reduced pressure, and the precipitate was filtered. The crude product was purified by reverse-phase chromatography (Biotage SNAP ULTRA C18, gradient from water to methanol). The fraction containing the product was concentrated under reduced pressure and stirred with a metal scavenger (2.5 g) at room temperature for 24 h. After removal of metal scavenger of SiliaMets by filtration, the solution was obtained by freeze-drying (893 mg, 55%).

Synthesis of *N*-butylacrylamide (ButylAAm)

Butyl amine (300 mg, 3.0 mmol) and *N,N*-diisopropylethylamine (0.63 mL, 3.6 mmol) were dissolved in dry dichloromethane (6 mL) and stirred in ice bath. Acryloyl chloride (0.29 mL, 3.6 mmol) was slowly dropped into the solution and the mixture was stirred for 10 h at room temperature. The progress of the reaction was confirmed by TLC (EtOAc : hexane = 2 : 1, UV). The reactant was washed by saturated brine once. The organic phase was dried by $MgSO_4$, filtered and concentrated under reduced pressure. The crude product was purified by silica column chromatography (EtOAc: hexane = 2 : 1) to give *N*-butyl acrylamide as white solid (203 mg, 53%).

^{1}H NMR (400 MHz, $CDCl_3$) δ in ppm: 6.28 (dd, J = 16.9, 1.4 Hz, 1H), 6.10 (dd, J = 16.9, 10.1 Hz, 1H), 5.64 (dd, J = 10.1, 1.4 Hz, 1H), 3.34 (q, J = 7.2 Hz, 2H), 1.53 (q, J = 7.2 Hz, 2H), 1.36 (m, J = 7.2 Hz, 2H), 0.94 (t, J = 7.2 Hz, 1H).

Synthesis of *N*-cyclohexyl acrylamide (CyHexAAm)

Cyclohexyl amine (292 mg, 2.9 mmol) and *N,N*-diisopropylethylamine (0.61 mL, 3.5 mmol) were dissolved in dry dichloromethane (5.2 mL) and stirred in ice bath. Acryloyl chloride (0.26 mL, 3.2 mmol) was slowly dropped into the solution and the mixture was stirred for 10 h at room temperature. The progress of the reaction was confirmed by TLC (EtOAc : hexane = 2 : 1, UV). The reactant was washed by saturated $NaHCO_3$(aq) once. The organic phase was dried by $MgSO_4$, filtered and concentrated under reduced pressure. The crude product was purified by silica column chromatography (EtOAc: hexane = 2: 1) to give *N*-cyclohexyl acrylamide (357 mg, 80%).

^{1}H NMR (400 MHz, $CDCl_3$) δ in ppm: 6.28 (dd, J = 16.8, 1.4 Hz, 1H), 6.10 (dd, J = 16.8, 10.0 Hz, 1H), 5.61 (dd, J = 10.0, 1.4 Hz, 1H), 3.85 (m, 1H), 1.96 (m, 2H), 1.73 (m, 2H), 1.39 (m, 2H), 1.17 (m, 3H).

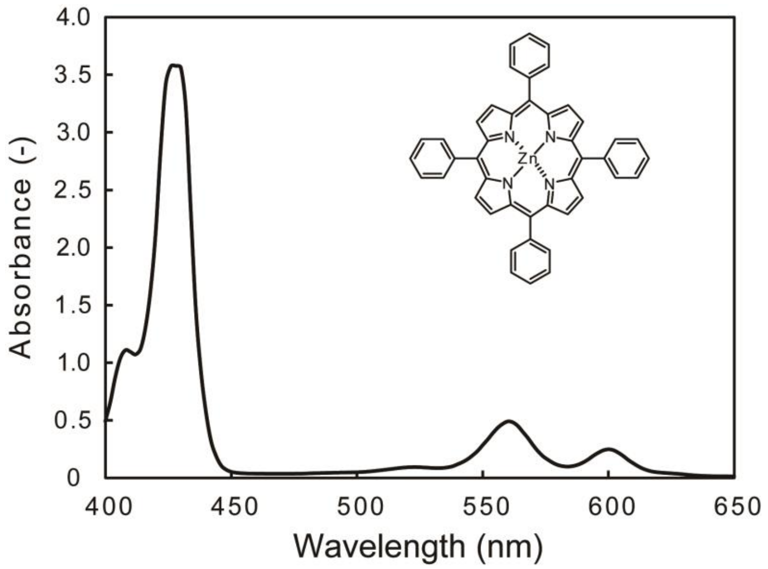

Figure S1. UV-vis absorbance spectra of ZnTPP in DMSO (0.74 mmol/L).

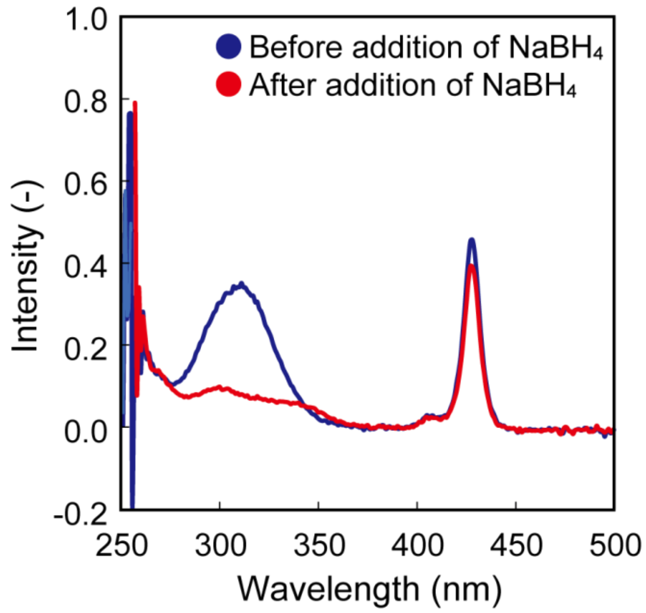

Figure S2. UV-vis absorbance spectra of the polymer (G0) in DMSO before (blue line) and after (red line) addition of $NaBH_4$. The absorbance peak at 430 nm is derived from ZnTPP in the polymerization solution.

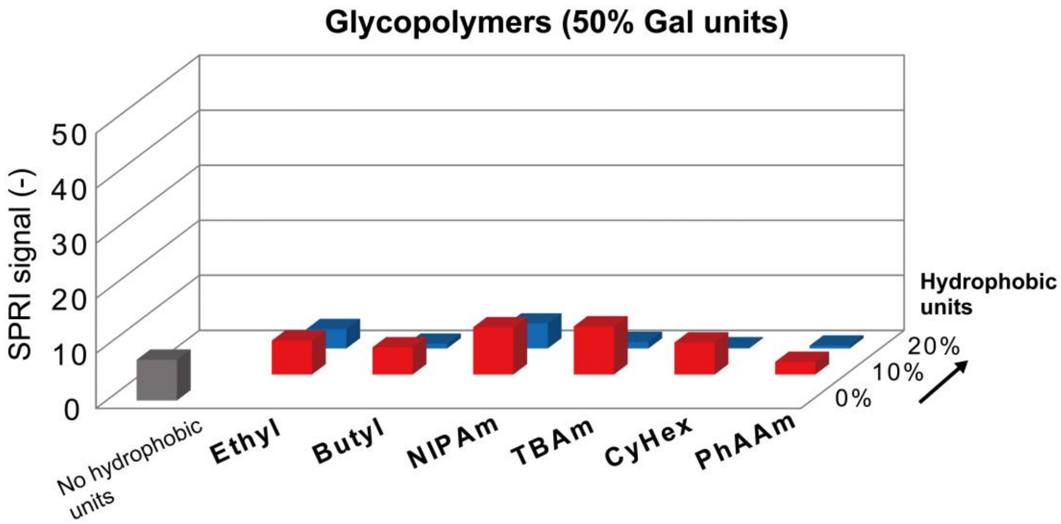

Figure S3. SPRI signals of the glycopolymer-immobilized surfaces with BSA (500 nM).

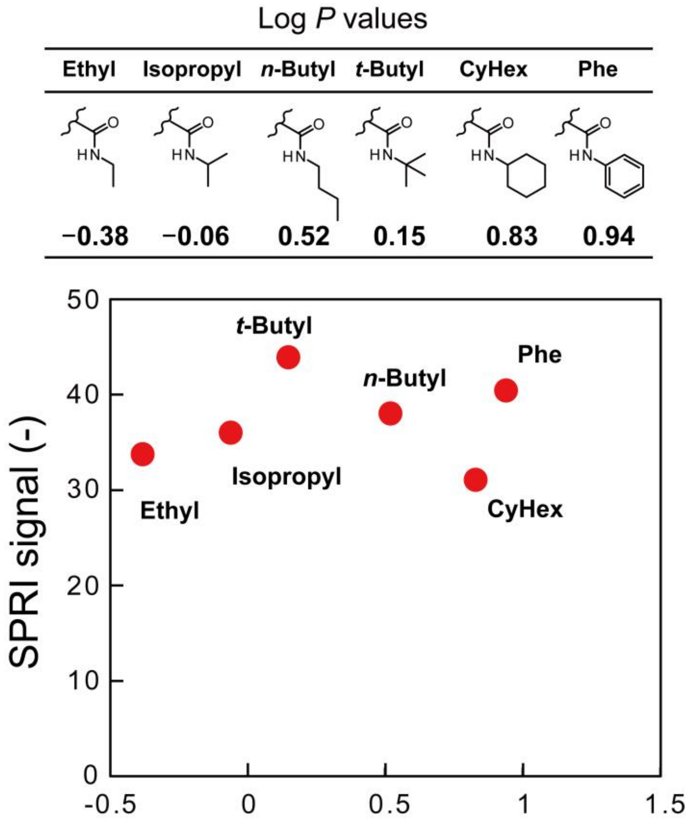

Figure S4. Plots of the SPRI signals of the glycopolymer-immobilized surface (10% hydrophobic units) for the log *P* values of the hydrophobic groups. The log *P* values were estimated using a ChemDraw software.

General Procedure of SPRI Measurement

SPRI gold chips were prepared by deposition of Cr (1.0 nm) and Au (50 nm) onto S-TIM35 glass substrate. SPRI gold chip was rinsed with EtOH and MilliQ, then dried by air blow. Glycopolymers were immobilized onto the gold spots by incubation of 10 g/L glycopolymer aqueous solution for over 3 h. Prior to the protein adsorption measurement, 10 mM Phosphate buffered saline (PBS) (pH 7.4, 137 mm NaCl, 2.68 mm KCl) was flew through (0.1 mL/min), and SPRI reflectivity change (defined as "SPRI signal") was monitored until the SPRI signal was stable. Then, protein solution with a certain concentration was injected with flow rate of 0.1 mL/min in all experiments, and the SPRI signal was monitored. In the measurement, the SPRI signal was regarded as the amount of protein adsorption. The binding constants of CTB were calculated with the Langmuir isotherm using the SPRI signals

$$\Delta R = \frac{K_a \, c \, \Delta R_{\max}}{1 + K_a \, c} \quad (1)$$

ΔR, ΔR_{max}, c, and K_a are the SPRI signal, the maximum SPRI signal, the protein concentration, and the binding constant, respectively. Based on eq 1, the plots of the SPRI signals were analyzed by nonlinear regression to derive the binding constants.

NMR spectra of the compounds

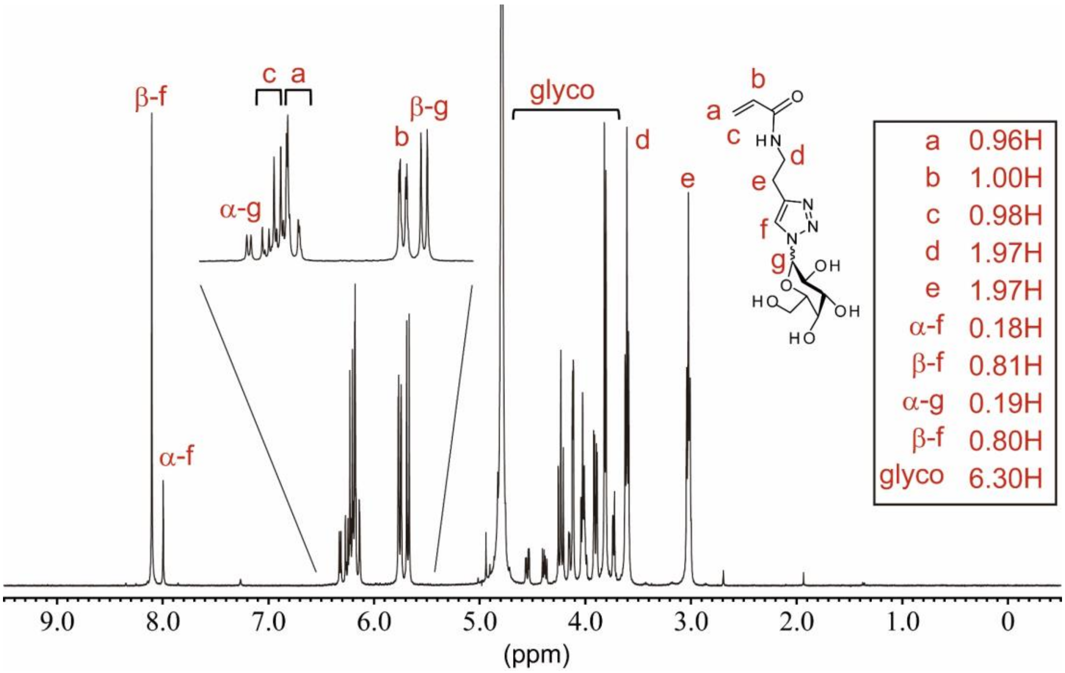

Figure S5. ^{1}H NMR spectrum of GalAAm (400 MHz, D_2O).

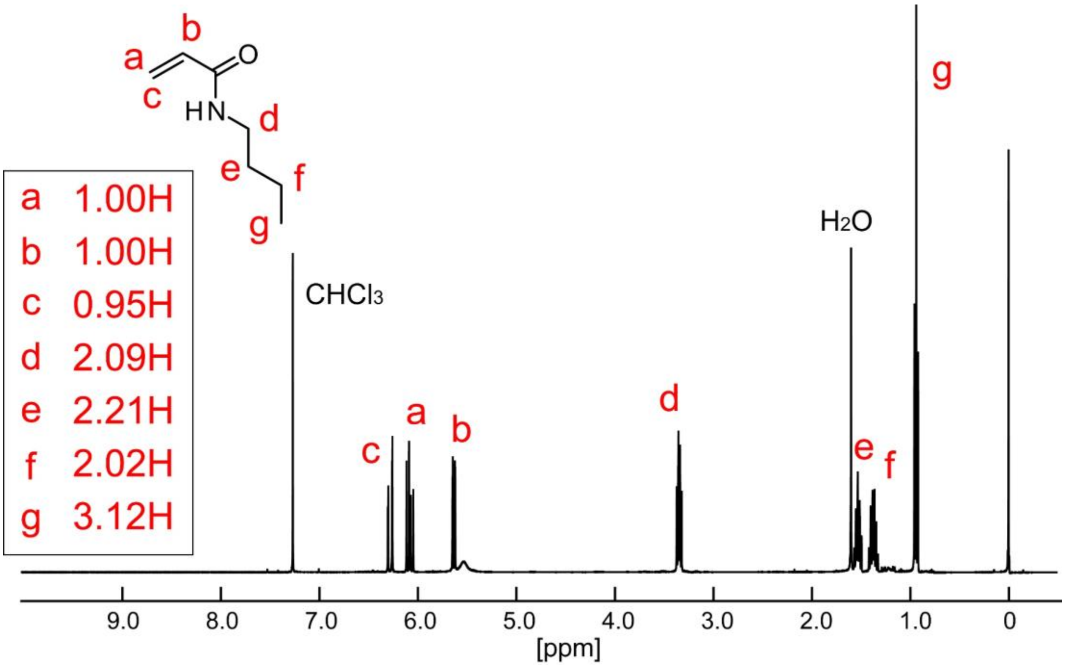

Figure S6. ^{1}H NMR spectrum of ButylAAm (400 MHz, $CDCl_3$).

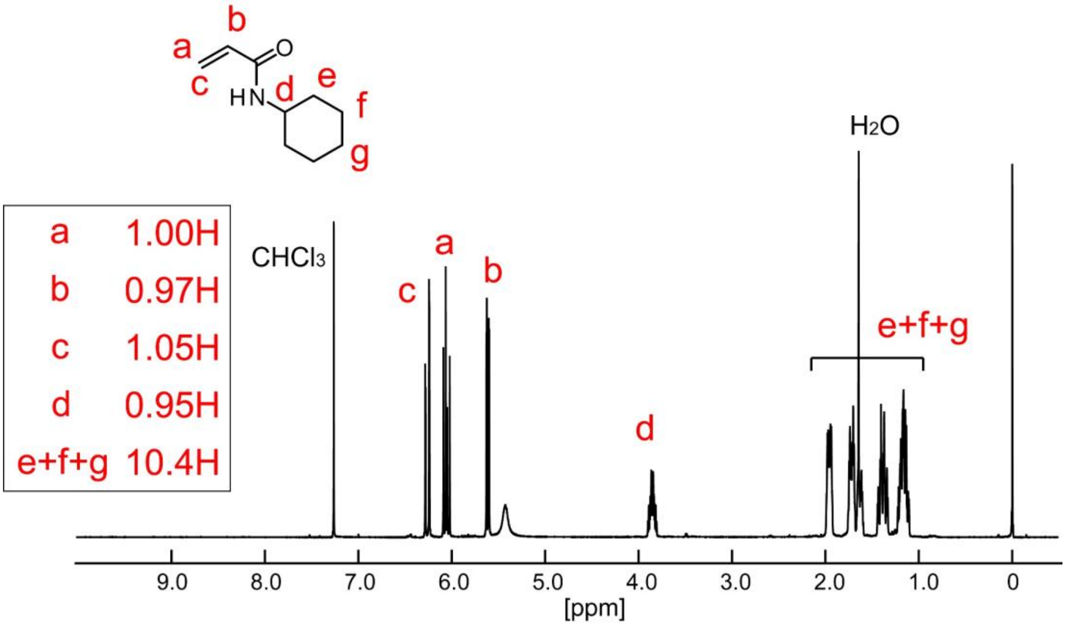

Figure S7. ^{1}H NMR spectrum of CyHexAAm (400 MHz, $CDCl_3$).

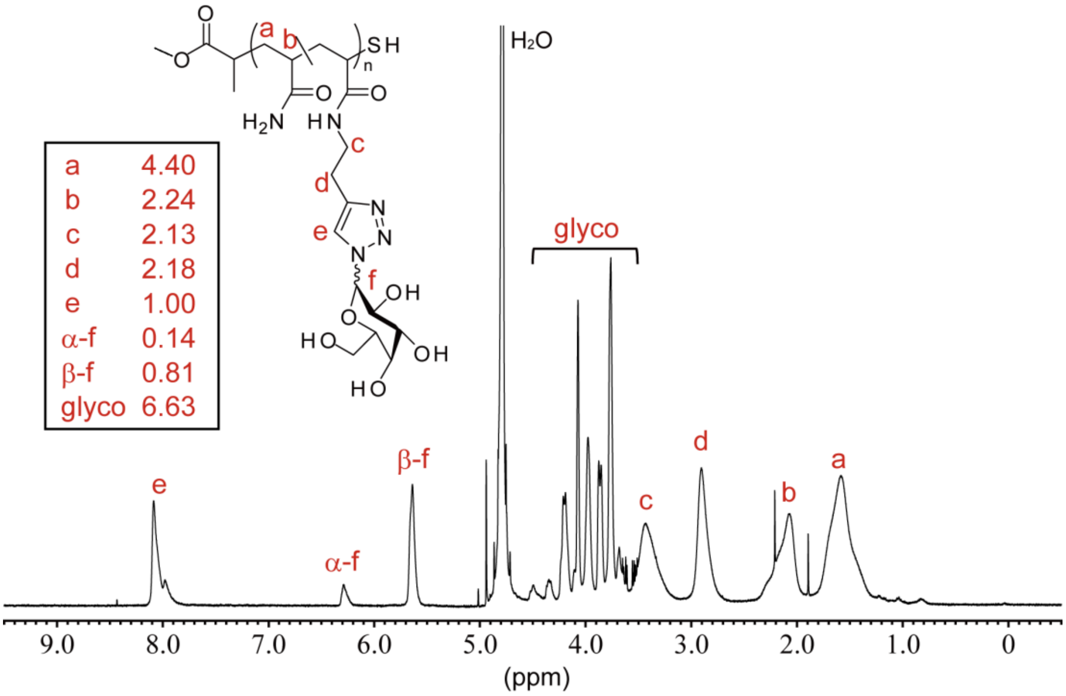

Figure S8. ^{1}H NMR spectrum of G50 (400 MHz, D_2O).

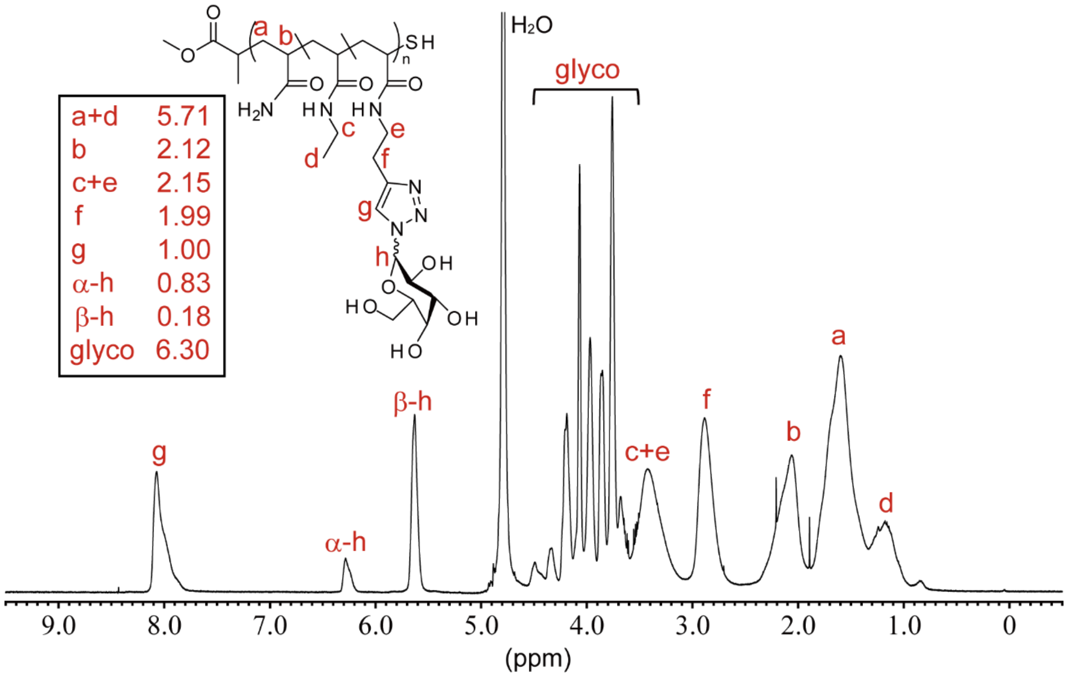

Figure S9. ^{1}H NMR spectrum of G50E10 (400 MHz, D_2O).

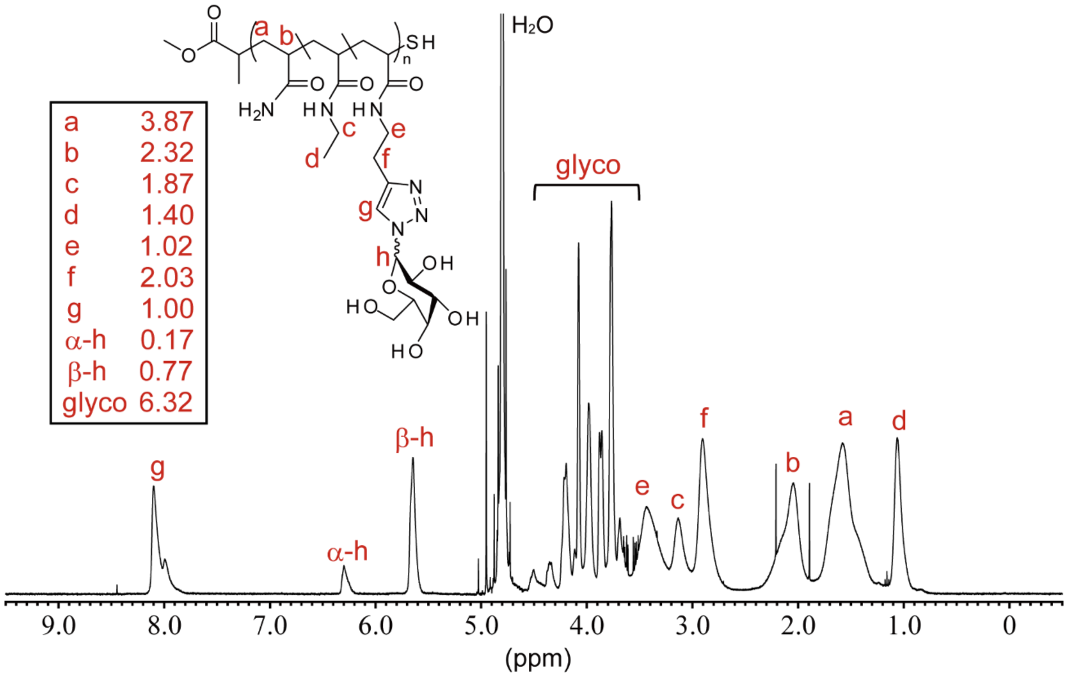

Figure S10. ^{1}H NMR spectrum of G50E20 (400 MHz, D_2O).

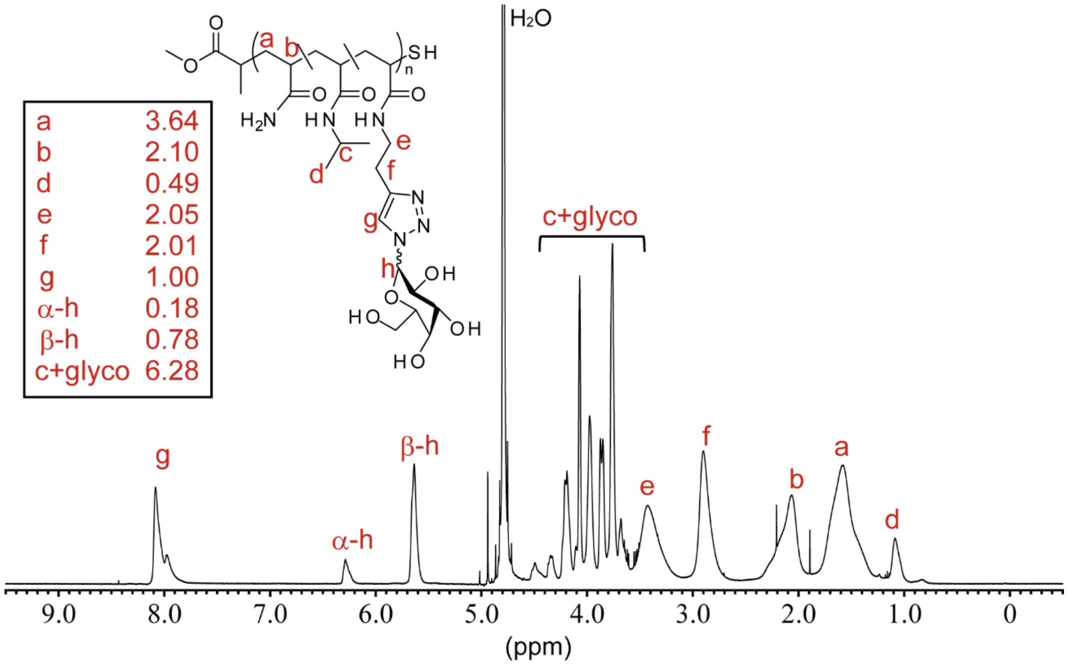

Figure S11. ^{1}H NMR spectrum of G50N10 (400 MHz, D_2O).

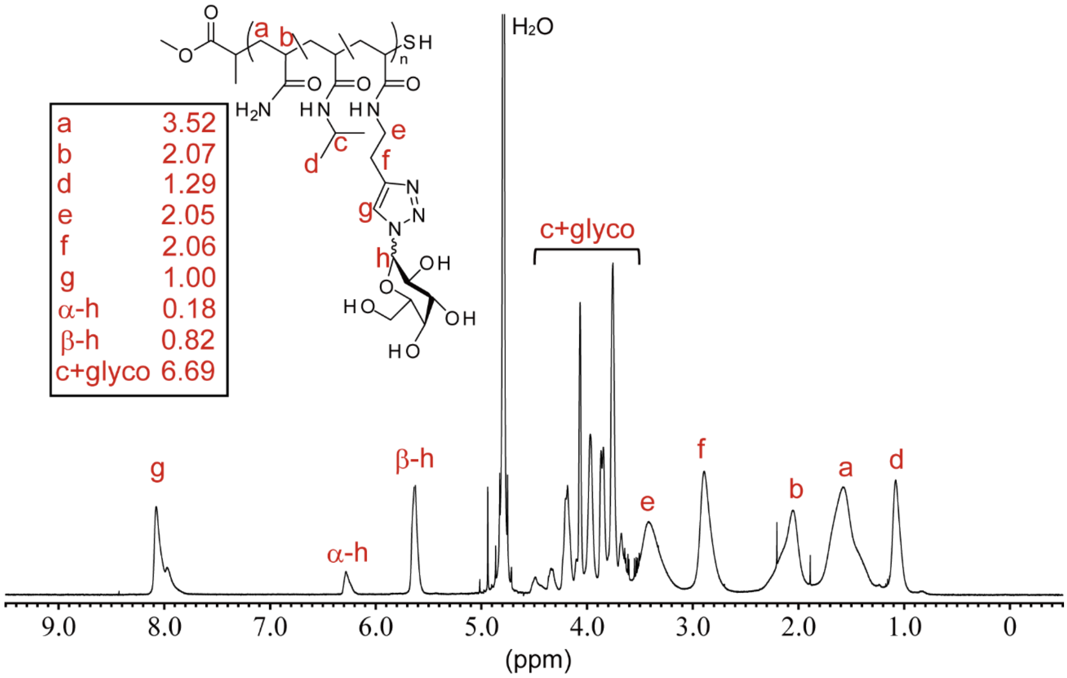

Figure S12. ^{1}H NMR spectrum of G50N20 (400 MHz, D_2O).

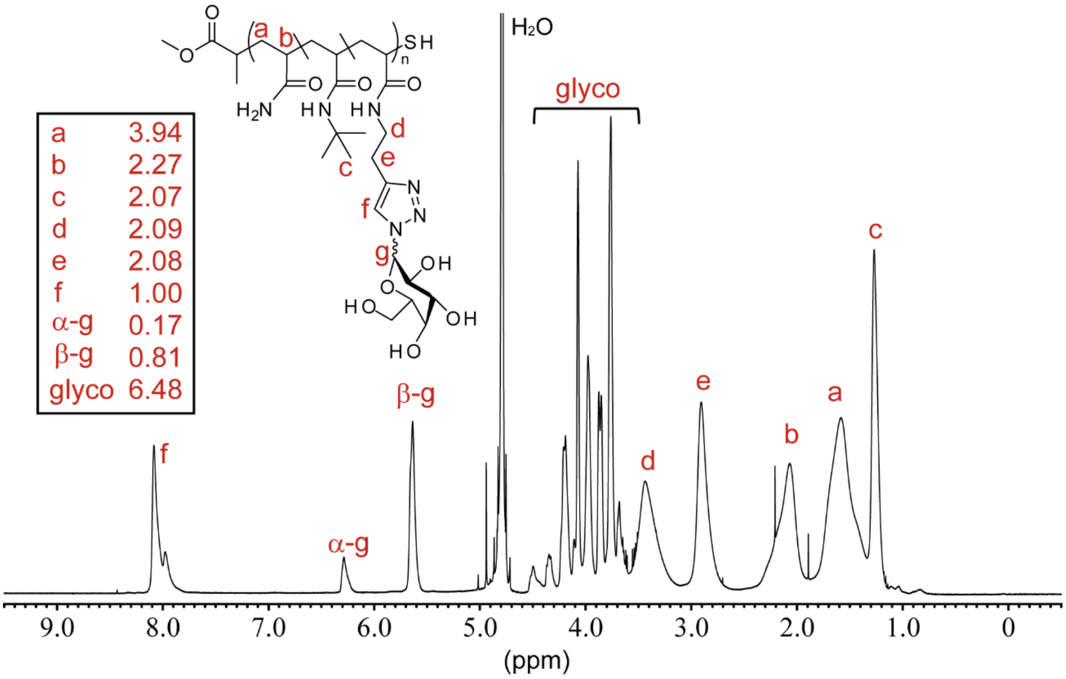

Figure S13. ^{1}H NMR spectrum of G50T10 (400 MHz, D_2O).

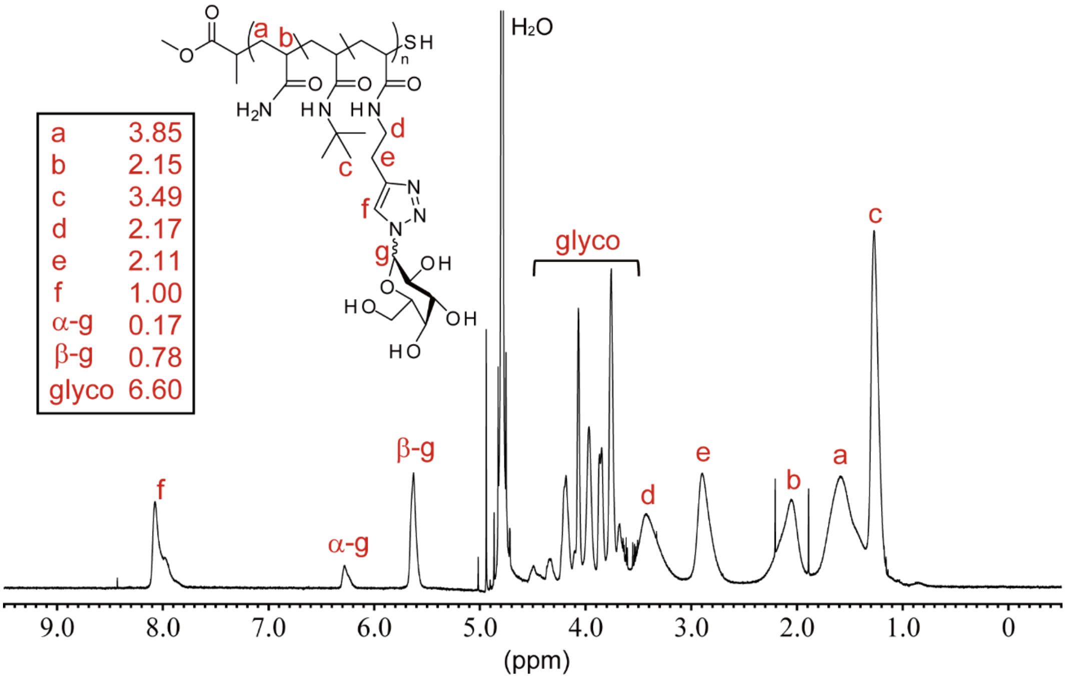

Figure S14. ^{1}H NMR spectrum of G50T20 (400 MHz, D_2O).

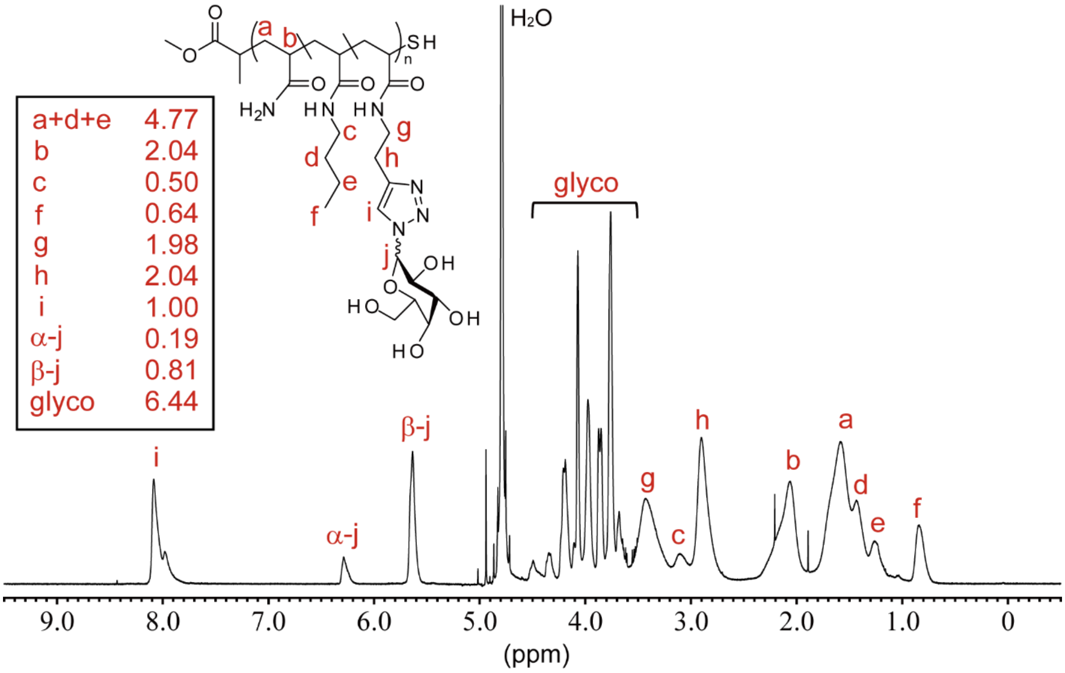

Figure S15. ^{1}H NMR spectrum of G50B10 (400 MHz, D_2O).

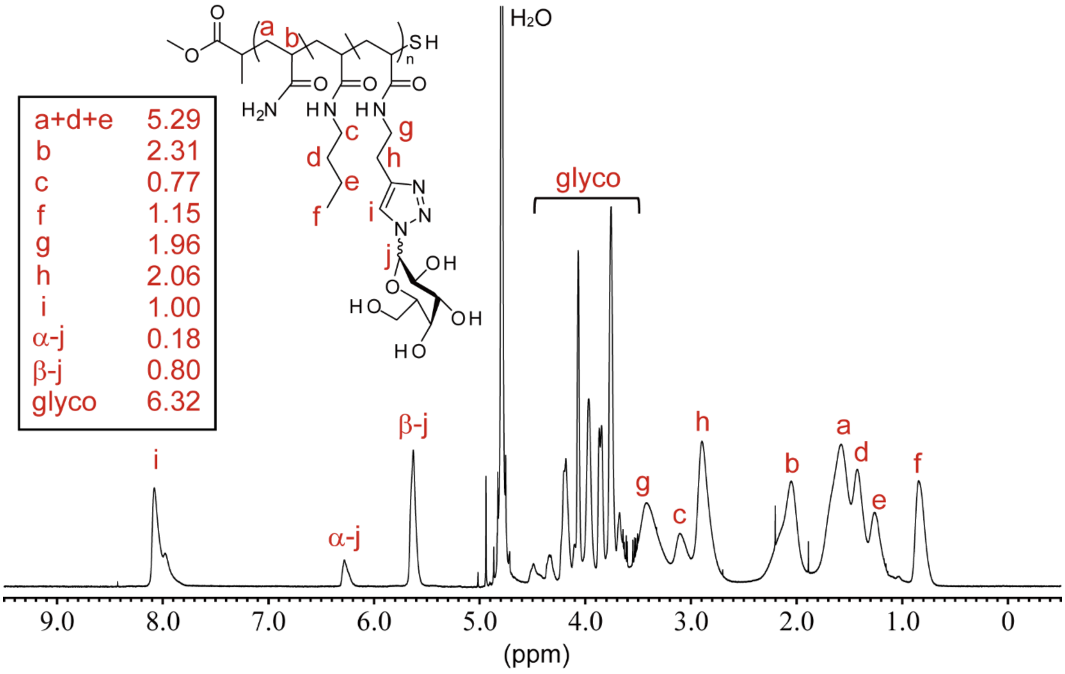

Figure S16. ^{1}H NMR spectrum of G50B20 (400 MHz, D_2O).

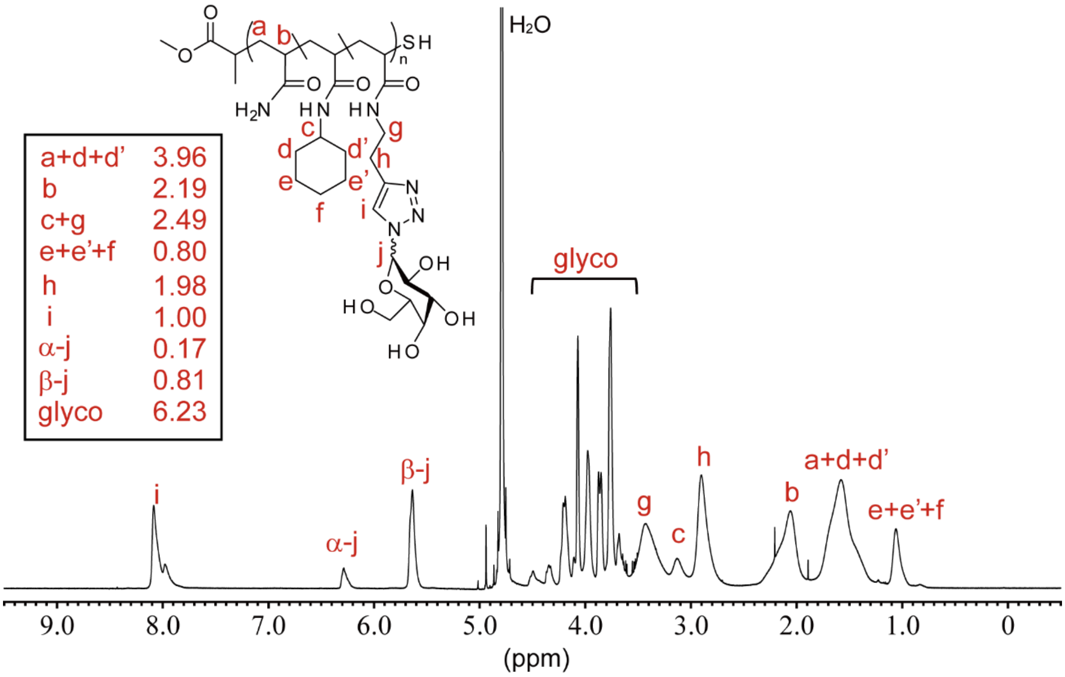

Figure S17. ^{1}H NMR spectrum of G50C10 (400 MHz, D_2O).

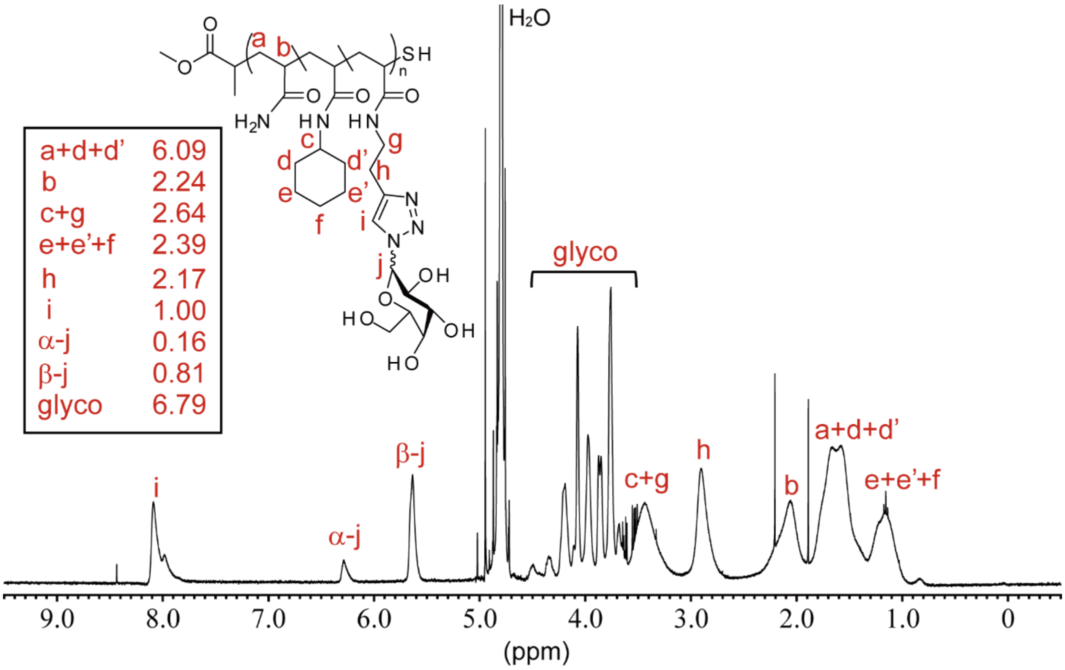

Figure S18. ^{1}H NMR spectrum of G50C20 (400 MHz, D_2O).

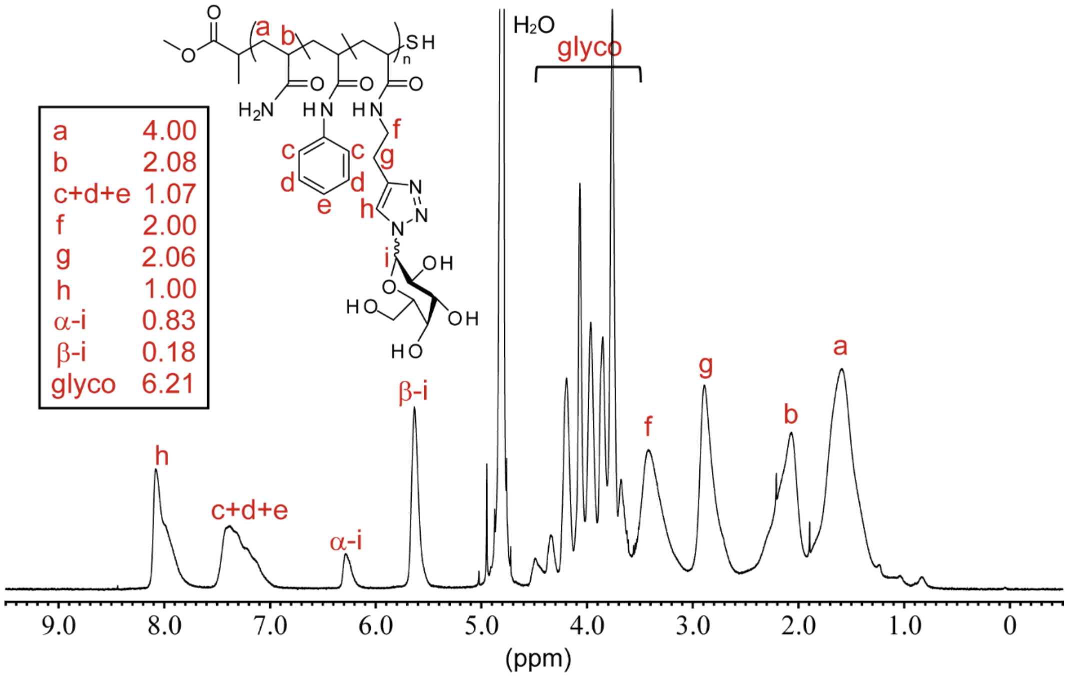

Figure S19. ^{1}H NMR spectrum of G50P10 (400 MHz, D_2O).

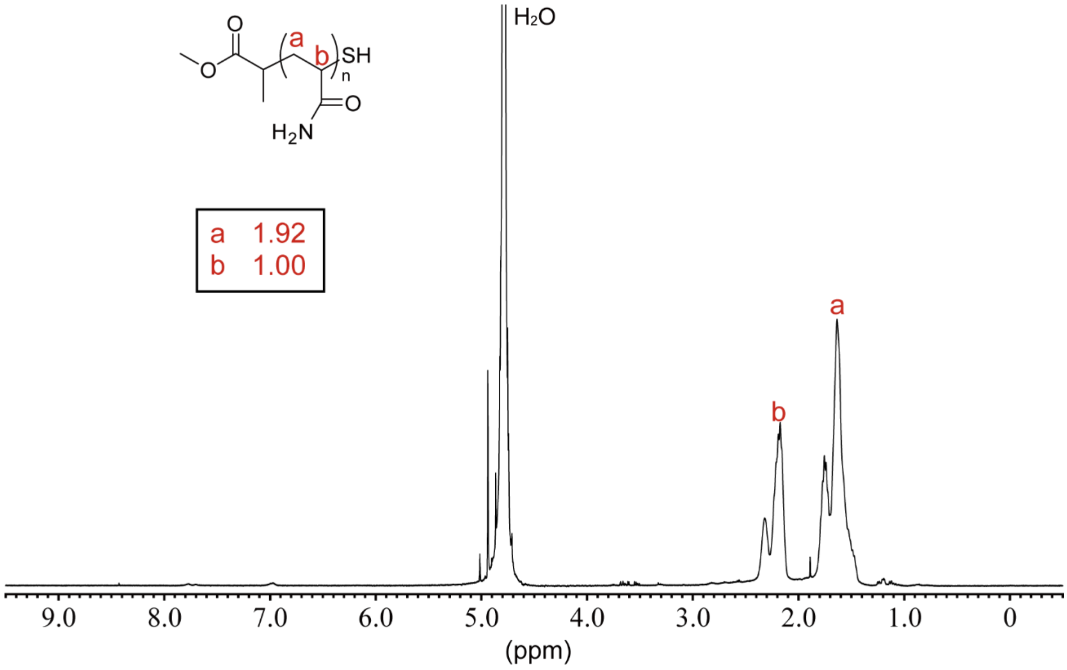

Figure S20. ^{1}H NMR spectrum of G0 (400 MHz, D_2O).

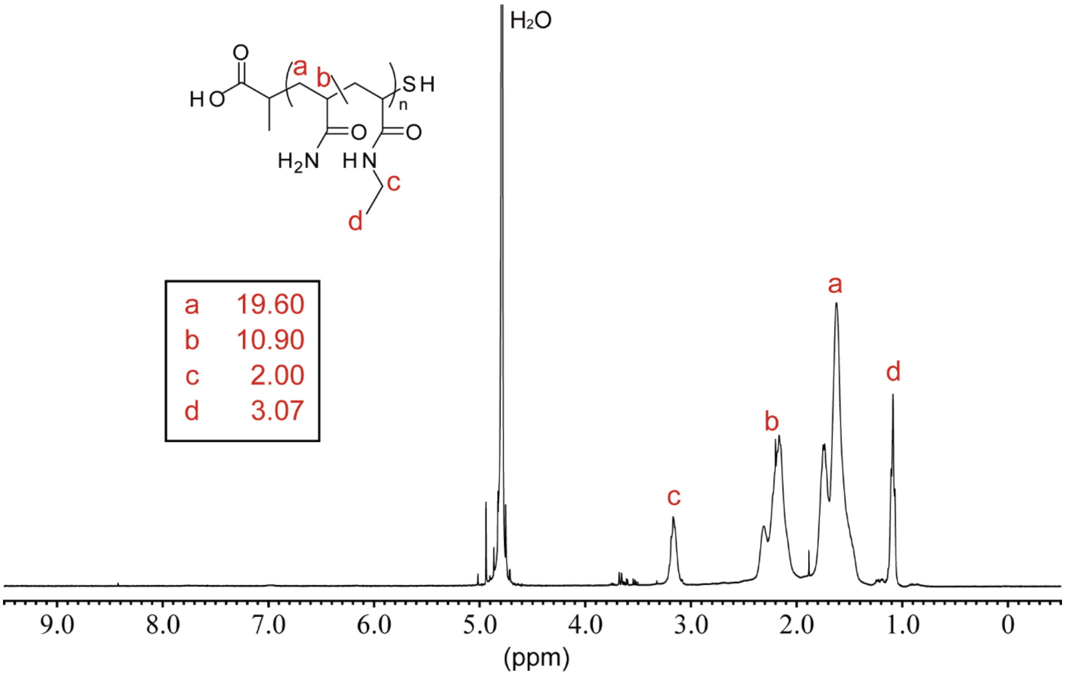

Figure S21. ^{1}H NMR spectrum of E10 (400 MHz, D_2O).

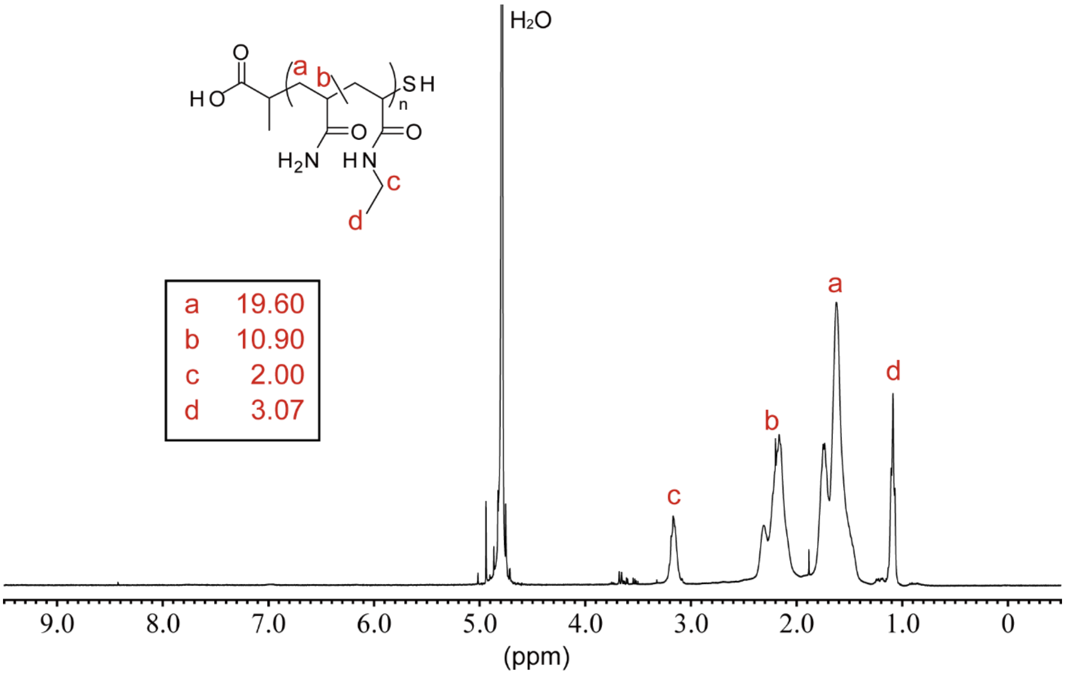

Figure S22. ^{1}H NMR spectrum of E20 (400 MHz, D_2O).

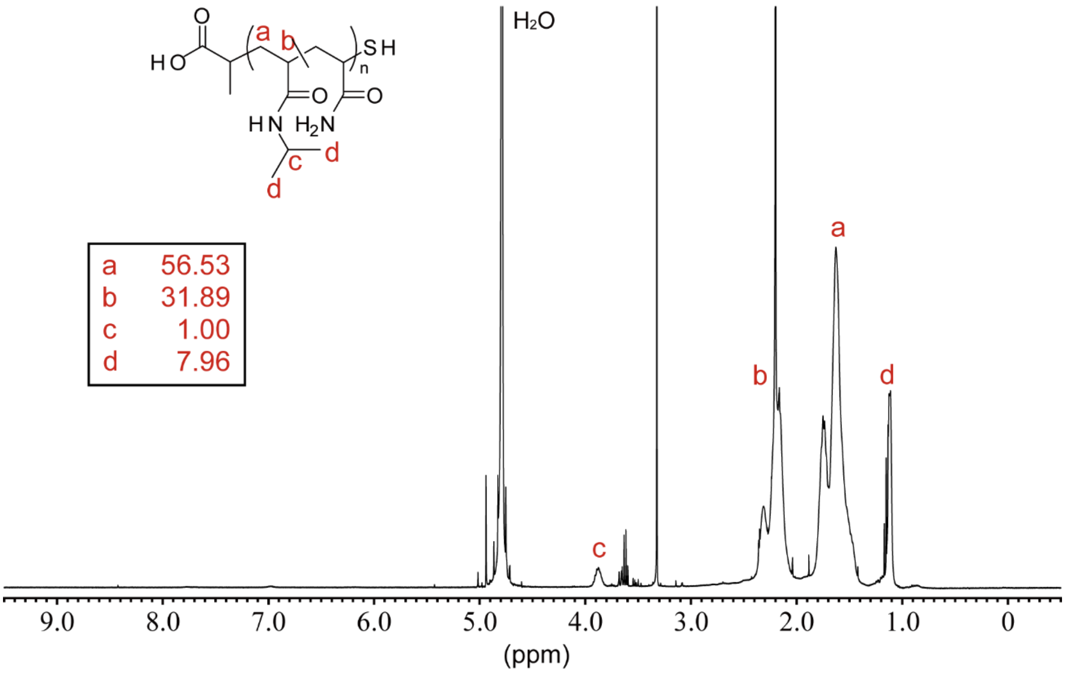

Figure S23. ^{1}H NMR spectrum of N10 (400 MHz, D_2O).

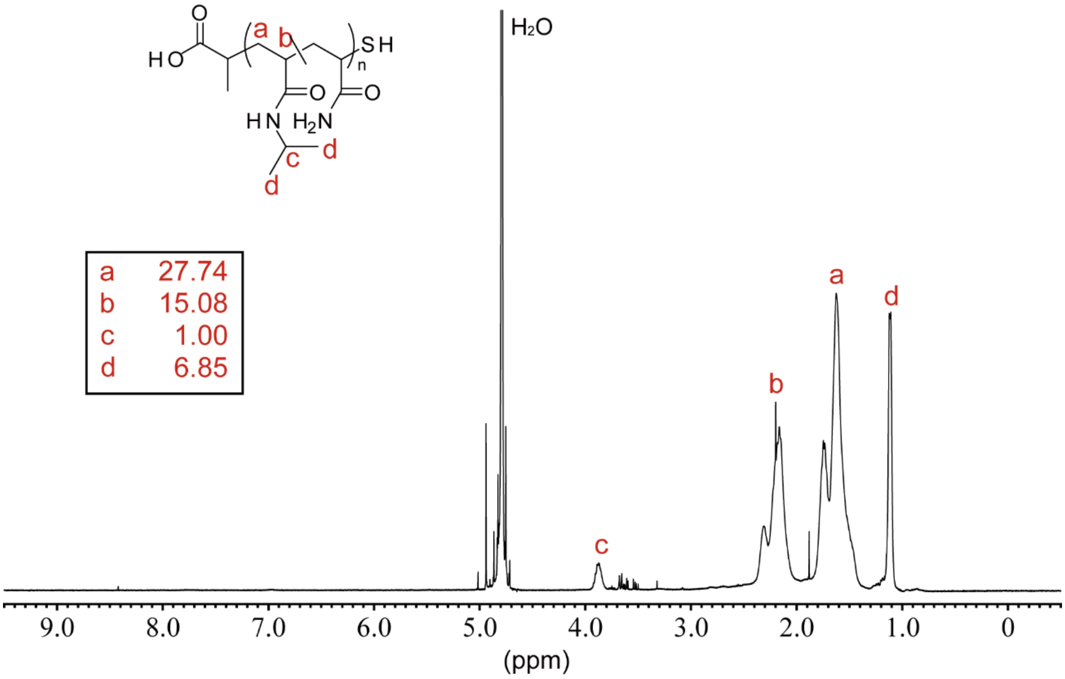

Figure S24. ^{1}H NMR spectrum of N20 (400 MHz, D_2O).

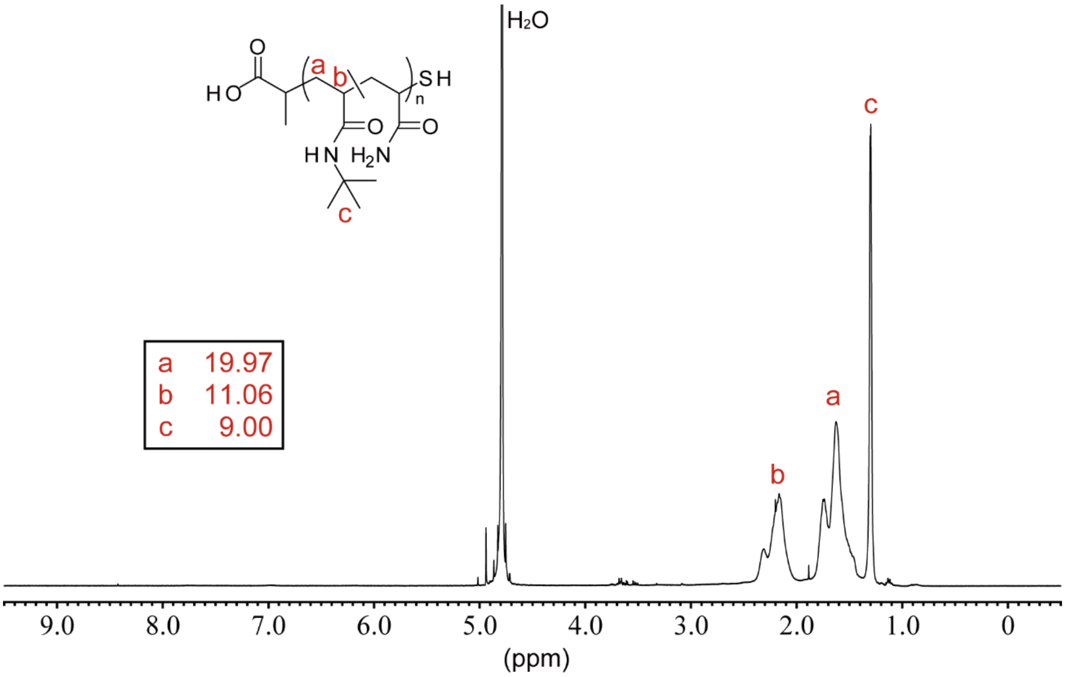

Figure S25. ^{1}H NMR spectrum of T10 (400 MHz, D_2O).

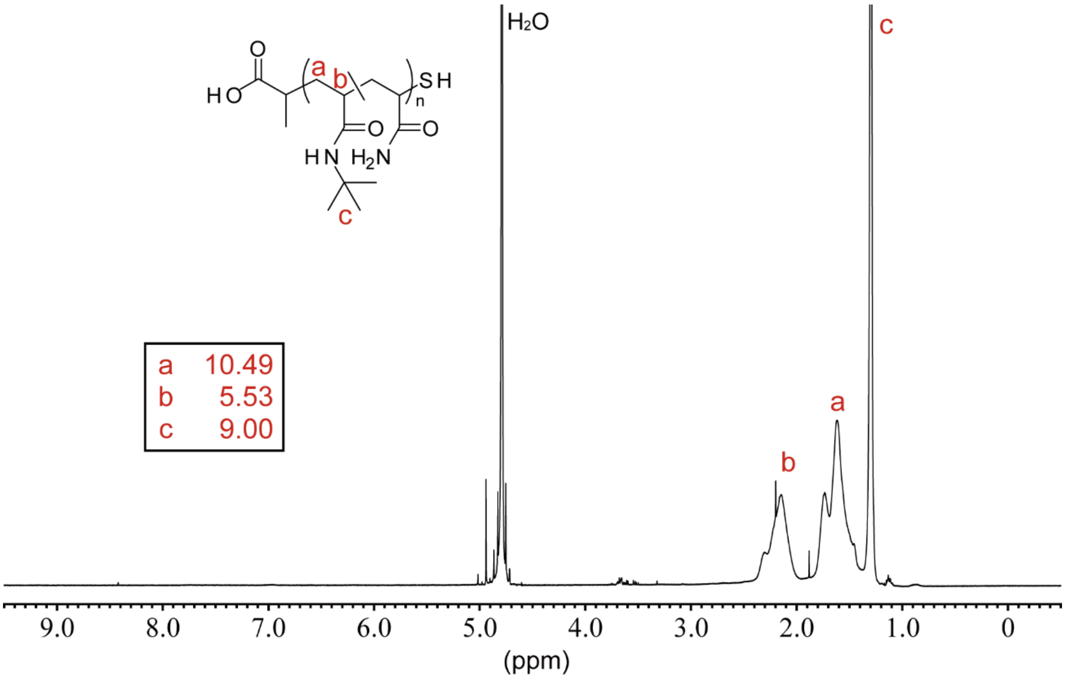

Figure S26. ^{1}H NMR spectrum of T20 (400 MHz, D_2O).

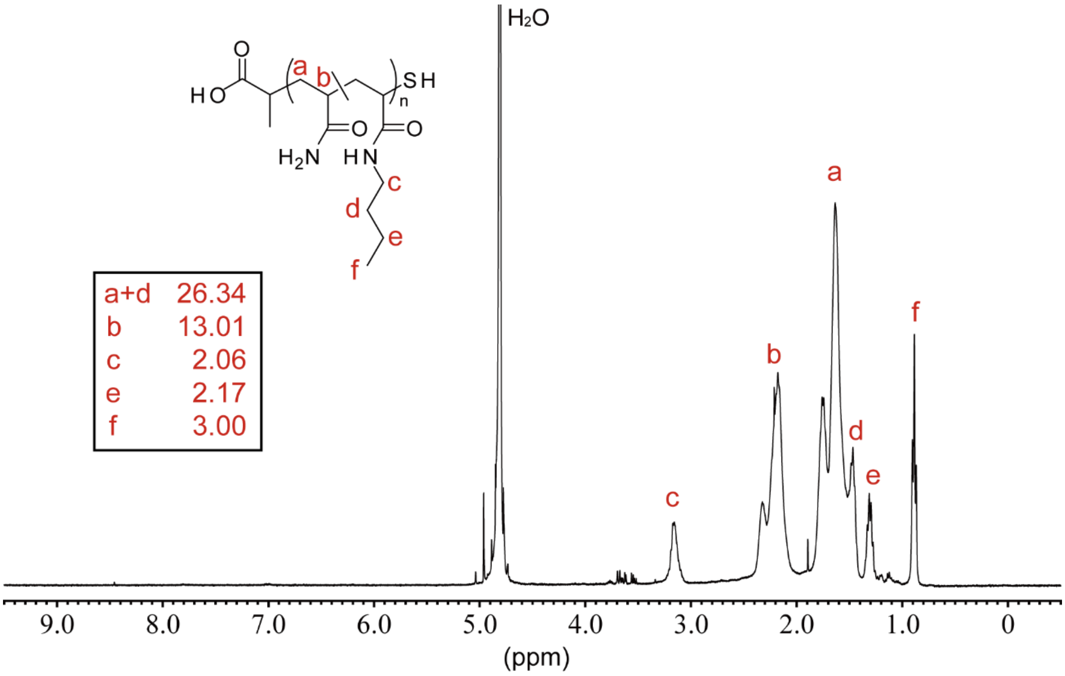

Figure S27. ^{1}H NMR spectrum of B10 (400 MHz, D_2O).

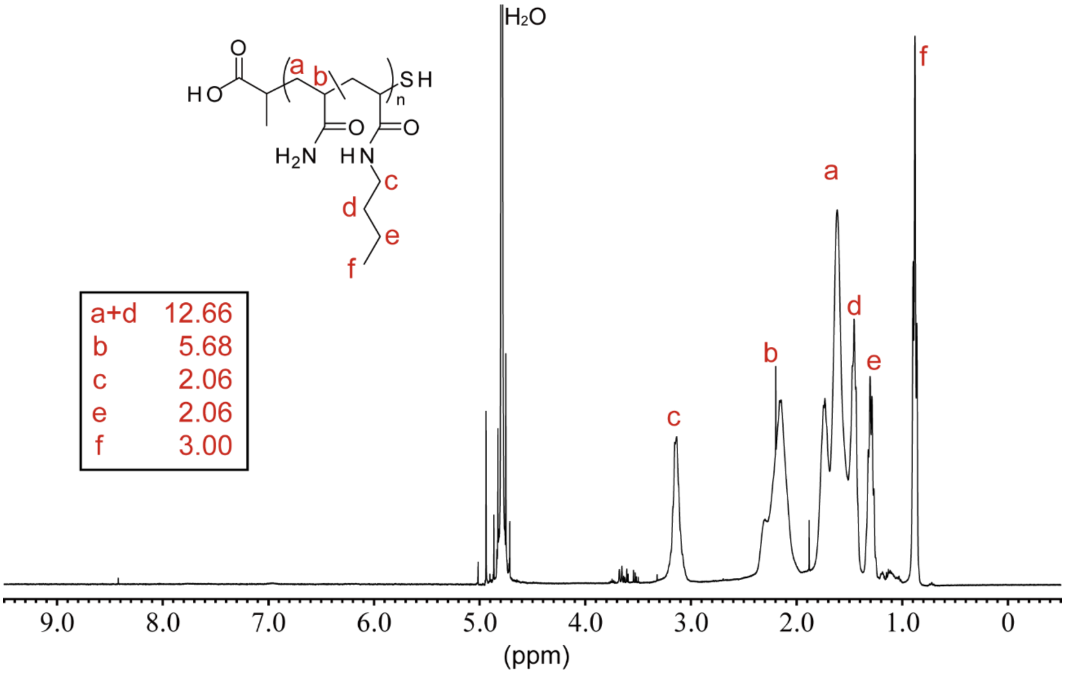

Figure S28. ^{1}H NMR spectrum of B20 (400 MHz, D_2O).

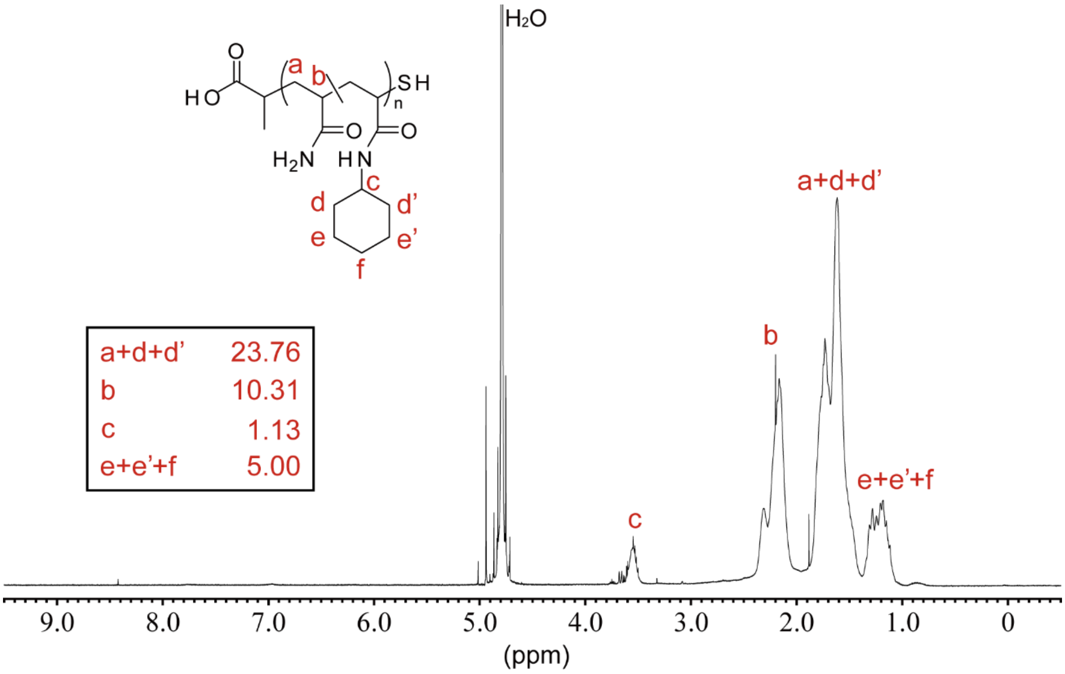

Figure S29. ^{1}H NMR spectrum of C10 (400 MHz, D_2O).

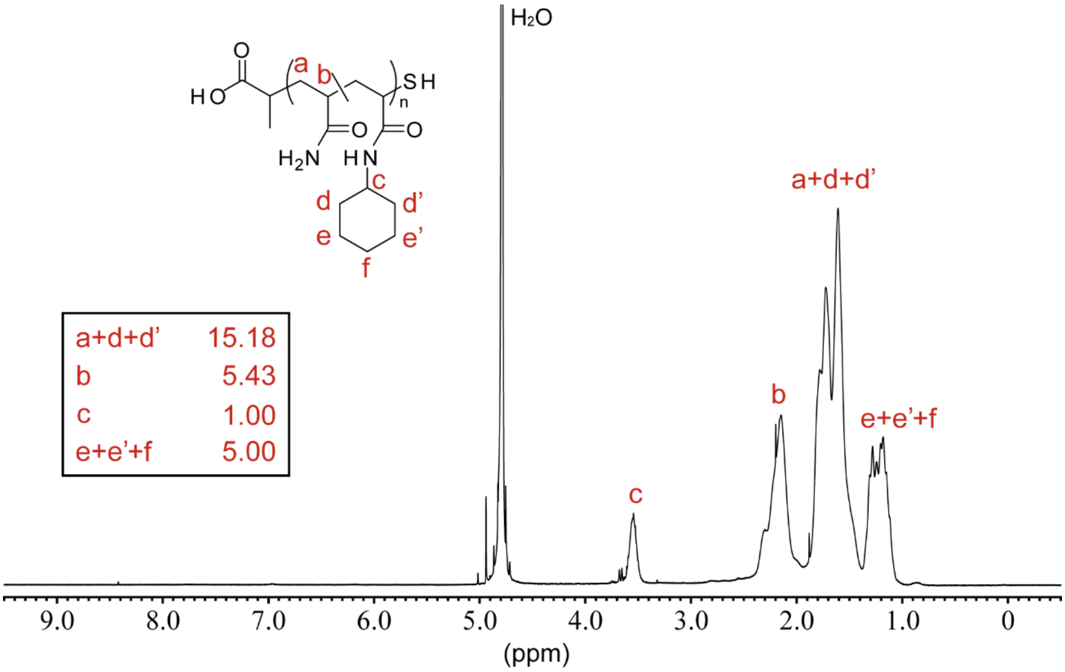

Figure S30. ^{1}H NMR spectrum of C20 (400 MHz, D_2O).

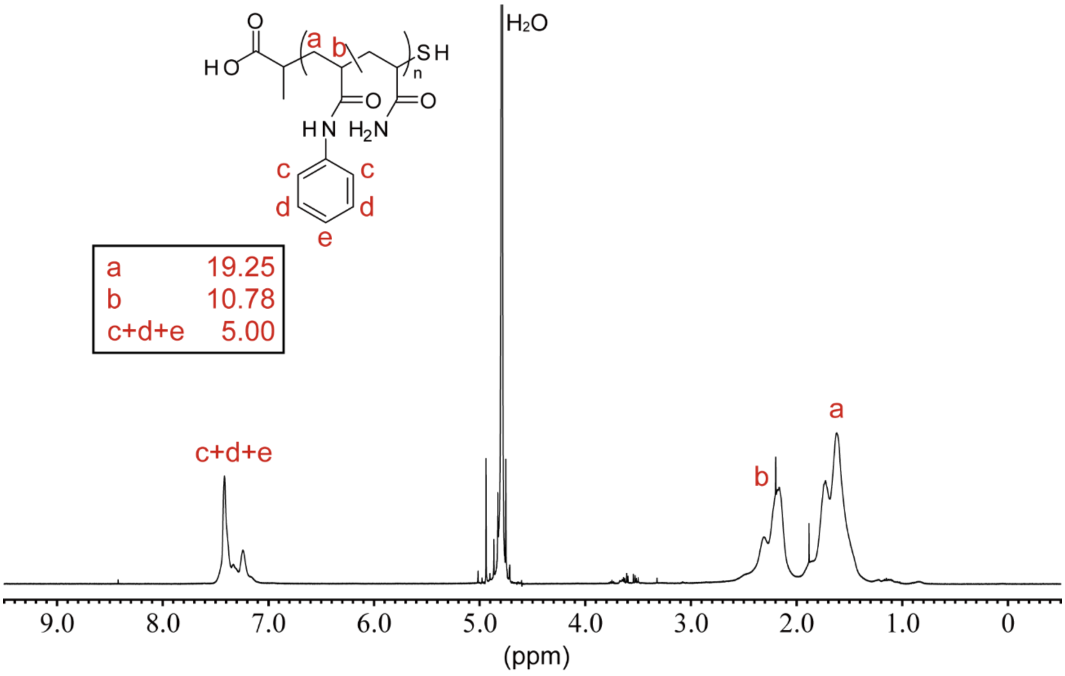

Figure S31. ^{1}H NMR spectrum of P10 (400 MHz, D_2O).

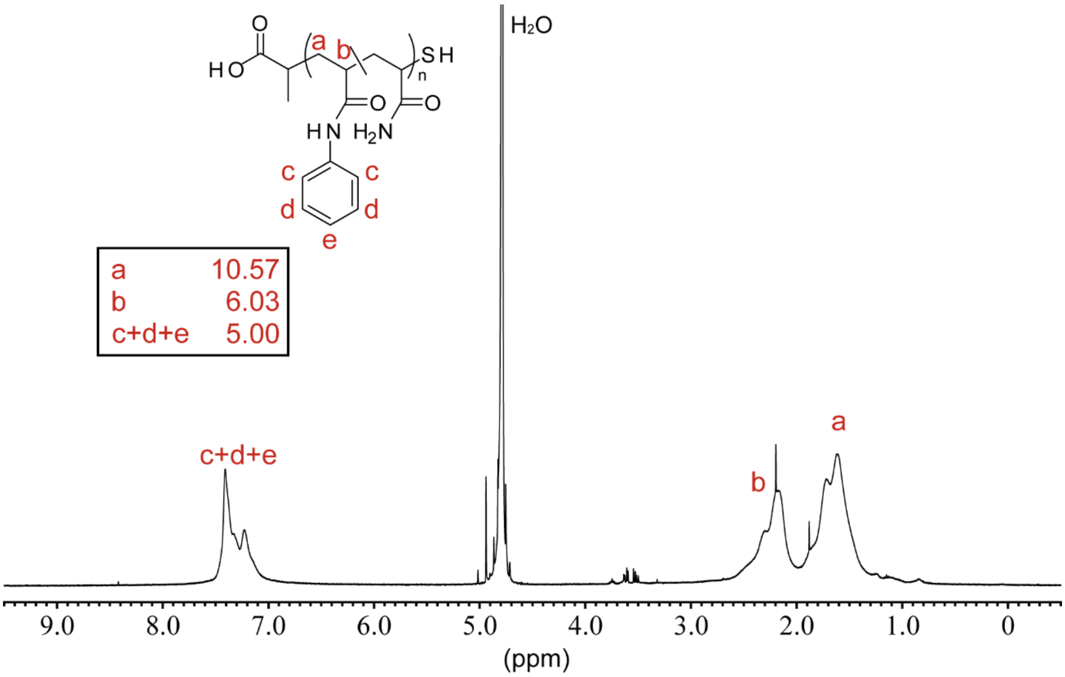

Figure S32. ^{1}H NMR spectrum of P20 (400 MHz, D_2O).

Reference

(1) Nagao, M.; Kurebayashi, Y.; Seto, H.; Takahashi, T.; Suzuki, T.; Hoshino, Y.; Miura, Y. Polyacrylamide backbones for polyvalent bioconjugates using "post-click" chemistry. *Polym Chem.*, 2016, *7*, 5920–5924.

(2) Tanaka, T.; Nagai, H.; Noguchi, M.; Kobayashi, A.; Shoda, S.-I. One-step conversion of unprotected sugars to β-glycosyl azides using 2-chloroimidazolinium salt in aqueous solution. *Chem. Commun.* **2009**, 3378–3379.

(3) Brassard, C. J.; Zhang, X.; Brewer, C. R.; Liu, P.; Clark, R. J.; Zhu, L. Cu(II)-Catalyzed Oxidative Formation of 5,5'-Bistriazoles. *J. Org. Chem.*, **2016**, *81*, 12091−12105.

(4) Nagao, M.; Fujiwara, Y.; Matsubara, T.; Hoshino, Y.; Sato, T.; Miura, Y. Design of Glycopolymers Carrying Sialyl Oligosaccharides for Controlling the Interaction with the Influenza Virus *Biomacromolecules*, **2017**, *18*, 4385−4392.
